# Associations Between Vascular Risk Factor Levels and Cognitive Decline Among Stroke Survivors

**DOI:** 10.1001/jamanetworkopen.2023.13879

**Published:** 2023-05-17

**Authors:** Deborah A. Levine, Bingxin Chen, Andrzej T. Galecki, Alden L. Gross, Emily M. Briceño, Rachael T. Whitney, Robert J. Ploutz-Snyder, Bruno J. Giordani, Jeremy B. Sussman, James F. Burke, Ronald M. Lazar, Virginia J. Howard, Hugo J. Aparicio, Alexa S. Beiser, Mitchell S. V. Elkind, Rebecca F. Gottesman, Silvia Koton, Sarah T. Pendlebury, Anu Sharma, Mellanie V. Springer, Sudha Seshadri, Jose R. Romero, Rodney A. Hayward

**Affiliations:** 1 Department of Internal Medicine and Cognitive Health Services Research Program, University of Michigan, Ann Arbor; 2 Department of Neurology and Stroke Program, University of Michigan, Ann Arbor; 3 Institute for Healthcare Policy and Innovation, University of Michigan, Ann Arbor; 4 Department of Nursing, University of Michigan, Ann Arbor; 5 Department of Epidemiology, Johns Hopkins Bloomberg School Public Health, Baltimore, Maryland; 6 Department of Physical Medicine and Rehabilitation, University of Michigan, Ann Arbor; 7 Department of Biostatistics, University of Michigan, Ann Arbor; 8 Department of Psychiatry and Michigan Alzheimer’s Disease Center, University of Michigan, Ann Arbor; 9 VA Ann Arbor Healthcare System, Ann Arbor, Michigan; 10 Department of Neurology, Ohio State University College of Medicine, Columbus; 11 Department of Neurology and Evelyn F. McKnight Brain Institute, Heersink School of Medicine, University of Alabama at Birmingham; 12 Department of Epidemiology, University of Alabama at Birmingham School of Public Health; 13 Department of Neurology, Boston University School of Medicine, Boston, Massachusetts; 14 Framingham Heart Study, National Heart, Lung, and Blood Institute, Framingham, Massachusetts; 15 Department of Biostatistics, Boston University School of Public Health, Boston, Massachusetts; 16 Department of Neurology, Vagelos College of Physicians and Surgeons, Columbia University, New York, New York; 17 Department of Epidemiology, Mailman School of Public Health, Columbia University, New York, New York; 18 Stroke Branch, National Institute of Neurological Disorders and Stroke, Bethesda, Maryland; 19 Department of Nursing, The Stanley Steyer School of Health Professions, Tel Aviv University, Tel Aviv, Israel; 20 Wolfson Centre for Prevention of Stroke and Dementia, Nuffield Department of Clinical Neurosciences, University of Oxford, Oxford, United Kingdom; 21 Department of Neurology and Glenn Biggs Institute for Alzheimer’s and Neurodegenerative Diseases, Joe R. and Teresa Lozano Long School of Medicine, University of Texas San Antonio; 22 NIHR Biomedical Research Centre, Departments of Medicine and Geratology, Oxford University Hospitals NHS Foundation Trust, Oxford, United Kingdom

## Abstract

**Importance:**

Incident stroke is associated with accelerated cognitive decline. Whether poststroke vascular risk factor levels are associated with faster cognitive decline is uncertain.

**Objective:**

To evaluate associations of poststroke systolic blood pressure (SBP), glucose, and low-density lipoprotein (LDL) cholesterol levels with cognitive decline.

**Design, Setting, and Participants:**

Individual participant data meta-analysis of 4 US cohort studies (conducted 1971-2019). Linear mixed-effects models estimated changes in cognition after incident stroke. Median (IQR) follow-up was 4.7 (2.6-7.9) years. Analysis began August 2021 and was completed March 2023.

**Exposures:**

Time-dependent cumulative mean poststroke SBP, glucose, and LDL cholesterol levels.

**Main Outcomes and Measures:**

The primary outcome was change in global cognition. Secondary outcomes were change in executive function and memory. Outcomes were standardized as *t* scores (mean [SD], 50 [10]); a 1-point difference represents a 0.1-SD difference in cognition.

**Results:**

A total of 1120 eligible dementia-free individuals with incident stroke were identified; 982 (87.7%) had available covariate data and 138 (12.3%) were excluded for missing covariate data. Of the 982, 480 (48.9%) were female individuals, and 289 (29.4%) were Black individuals. The median age at incident stroke was 74.6 (IQR, 69.1-79.8; range, 44.1-96.4) years. Cumulative mean poststroke SBP and LDL cholesterol levels were not associated with any cognitive outcome. However, after accounting for cumulative mean poststroke SBP and LDL cholesterol levels, higher cumulative mean poststroke glucose level was associated with faster decline in global cognition (−0.04 points/y faster per each 10–mg/dL increase [95% CI, −0.08 to −0.001 points/y]; *P* = .046) but not executive function or memory. After restricting to 798 participants with apolipoprotein E4 (APOE4) data and controlling for APOE4 and APOE4 × time, higher cumulative mean poststroke glucose level was associated with a faster decline in global cognition in models without and with adjustment for cumulative mean poststroke SBP and LDL cholesterol levels (−0.05 points/y faster per 10–mg/dL increase [95% CI, −0.09 to −0.01 points/y]; *P* = .01; −0.07 points/y faster per 10–mg/dL increase [95% CI, −0.11 to −0.03 points/y]; *P* = .002) but not executive function or memory declines.

**Conclusions and Relevance:**

In this cohort study, higher poststroke glucose levels were associated with faster global cognitive decline. We found no evidence that poststroke LDL cholesterol and SBP levels were associated with cognitive decline.

## Key Points

**Question:**

Are higher blood pressure, glucose, and low-density lipoprotein cholesterol in stroke survivors associated with cognitive decline?

**Findings:**

In this cohort study using a meta-analysis of individual participant data from 982 stroke survivors from 4 cohort studies, higher cumulative mean poststroke glucose level, but not blood pressure or low-density lipoprotein cholesterol levels, was associated with faster decline in global cognition.

**Meaning:**

These findings suggest that higher cumulative glucose levels may contribute to faster cognitive decline in stroke survivors, representing a potential treatment target to preserve cognition after stroke.

## Teaser

This cohort study evaluates associations of poststroke systolic blood pressure, glucose, and low-density lipoprotein cholesterol levels with cognitive decline among survivors of stroke.

## Introduction

Incident stroke is associated with accelerated, persistent cognitive decline.^1^ Stroke survivors are as much as 50 times more likely than stroke-free adults to develop dementia, with as many as 35% developing dementia within 1 year.^2^ Among stroke survivors, as much as 53% of dementia risk is attributable to stroke.^3^ Preventing or delaying cognitive decline and dementia could lead to better survival,^4^ functioning,^5,6^ and quality of life^7^ in survivors.

High blood pressure (BP), glucose, and low-density lipoprotein (LDL) cholesterol levels are associated with cognitive decline and dementia in stroke-free adults^8,9,10^ and are risk factors for stroke. It is unclear whether poststroke levels of these modifiable vascular risk factors (VRFs) are associated with cognitive decline, independent of prestroke VRF and cognition levels. As many as 85% of stroke survivors have high BP, 40% have diabetes, and 60% have dyslipidemia.^11^ Prior studies have been limited by lack of longitudinal cognitive and VRF measurements before and after stroke hospitalization, small sample size, and clinical samples. Evidence on the association of poststroke VRF levels with cognitive decline is needed to help clinicians individualize treatment and researchers identify targets for interventions to preserve cognitive function after stroke.

The Effect of Vascular Risk Factors on Cognitive Trajectories after Stroke (STROKE COG) study is an individual patient data (IPD) meta-analysis of cohort studies that combines high-quality longitudinal data with detailed cognitive assessments, objective measures of VRFs, and physician-adjudicated incident stroke. This article tests the hypothesis that higher poststroke systolic BP (SBP), glucose, or LDL cholesterol levels are associated with faster cognitive decline.

## Methods

### Study Design, Participants, and Measurements

Following reporting guideline for IPD meta-analyses,^12^ STROKE COG pooled individual participant data from 4 US prospective cohort studies: Atherosclerosis Risk in Communities Study (ARIC),^13^ Cardiovascular Health Study (CHS),^14^ Framingham Offspring Study (FOS),^15^ and Reasons for Geographic and Racial Differences in Stroke Study (REGARDS).^16^ We included data from cohort enrollment through December 31, 2019 (eMethods in Supplement 1). We chose these cohorts because they had at least 50 participants with physician-adjudicated incident stroke (ischemic or hemorrhagic) and objective measures of BP and cognition before and after.^17^

We included participants aged 18 years or older who had an incident stroke during the cohorts’ follow-up period, at least 1 prestroke cognitive assessment, at least 1 poststroke cognitive assessment, at least 1 prestroke SBP measurement, and at least 1 poststroke SBP measurement before or at the last poststroke cognitive assessment. We excluded participants reporting stroke history at baseline,^1^ with cohort-defined dementia at or before the incident stroke (eMethods in Supplement 1), as well as participants reporting race other than White or Black due to differences in cohort design.

The University of Michigan institutional review board approved the study. Participating institutions’ review boards approved the cohort studies. Participants provided written informed consent.

### Cognitive Function Assessments

Trained cohort staff administered cognitive tests consistent with the Vascular Cognitive Impairment Harmonization Standards^18^ to participants in-person (ARIC, CHS, FOS) or by telephone (REGARDS) using standardized protocols. Telephone assessments of global cognition, executive function, and memory tests were performed using validated tests. These domains can be measured reliably and validly over the telephone.^19^

To make inferences about cognitive domains instead of individual test items and resolve the challenge of different tests administered across the cohorts, we cocalibrated available cognitive test items common across cohorts and unique to individual cohorts into 3 factors (domains) representing global cognition (global cognitive performance), memory (learning and delayed recall or recognition), and executive function (complex and/or speeded cognitive functions) using item response theory methods and confirmatory factor analysis.^20,21,22,23^ Cognitive factor scores were estimated using regression models based on the method in Mplus version 8 (Muthén & Muthén).^24,25^ Factor scores have the same meaning across cohorts and were set to a *t * score metric (mean [SD], 50 [10]) at a participant’s first cognitive assessment; a 1-point difference represents a 0.1-SD difference in the distribution of cognition across the cohorts. Higher cognitive scores indicate better performance (eMethods in Supplement 1). The primary outcome was change in global cognition. Secondary outcomes were change in executive function and memory.

### Measurement of BP, Glucose, and LDL Cholesterol

Each cohort study measured BP, glucose, and LDL cholesterol before and after stroke at in-person visits using standard protocols and equipment. The 4 cohort studies instructed participants to fast before exams, except for CHS exam 7. Poststroke SBP, glucose, and LDL cholesterol levels were the measurements of interest and were summarized as the time-dependent cumulative means (ie, running averages) of all measurements at or before each poststroke cognitive assessment based on prior research (eMethods in Supplement 1).^9,23,26,27^ Prestroke SBP, glucose, and LDL cholesterol levels were summarized as arithmetic means of all values before stroke and treated as baseline covariates.

### Covariates

Covariates were selected based on a conceptual model^28^ and available cohort measures. Age was at the time of stroke. Sex, self-identified race, and cohort study were measured at cohort baseline. Prestroke SBP, glucose, LDL cholesterol, and harmonized global cognition, executive function, and memory scores were summarized as arithmetic means of all measurements before the stroke. Education, income, cigarette smoking, body mass index (calculated as weight in kilograms divided by height in meters squared), waist circumference, physical activity, alcohol use, histories of myocardial infarction^29^ and atrial fibrillation, glomerular filtration rate,^30^ number of apolipoprotein E4 (APOE4) alleles, and antihypertensive, antihyperglycemic, lipid-lowering medication were measured using the values closest to and before, but not after, the first poststroke cognitive assessment. Poststroke depressive symptoms were measured by the Center for Epidemiologic Studies Depression Scale^31,32^ and summarized as time-invariant arithmetic means of all poststroke measurements. The eMethods in Supplement 1 describes covariate details.

### Statistical Analysis

We performed a complete case analysis excluding a small number of participants (138 of 1120 [12.3%]) due to missing covariate data. The eFigure in Supplement 1 shows the cohort derivation. We used linear mixed-effects models to estimate longitudinal changes in each continuous cognitive outcome. We also performed within-cohort analyses.

Models included the covariates in Table 1, follow-up time, participant-specific random effects for intercepts and slopes, and 2-way interaction terms involving follow-up time crossed with sex and age at the time of stroke and antihypertensive, antihyperglycemic, lipid-lowering medication use. Follow-up time was treated as a continuous measure, defined as years since stroke. There was no evidence of a significant race × follow-up time interaction on cognitive trajectories or quadratic effects of time, poststroke SBP, glucose, or LDL cholesterol.

**Table 1.** Characteristics of Participants

Variable	Stroke survivors, No. (%) (N = 982)
Age at time of stroke, y	
Range	44.1-96.4
Median (IQR)	74.6 (69.1-79.8)
Measures at cohort baseline	
Sex	
Female	480 (48.9)
Male	502 (51.1)
Race^a^	
Black	289 (29.4)
White	693 (70.6)
Cohort	
ARIC	238 (24.2)
CHS	332 (33.8)
FOS	101 (10.3)
REGARDS	311 (31.7)
Measures by the first poststroke cognitive assessment	
Education	
<High school	176 (18.0)
High school	285 (29.0)
Some college	230 (23.4)
≥College graduate	291 (29.6)
Income	
<$5000	28 (2.9)
$5000-$24 999	296 (30.1)
$25 000-$34 999	163 (16.6)
$35 000-$49 999	139 (14.1)
≥$50 000	206 (21.0)
Refused to answer or missing	150 (15.3)
Current cigarette smoking	105 (10.7)
Any physical activity	727 (74.0)
Body mass index, median (IQR)^b^	27.2 (24.6-30.5)
Waist circumference, median (IQR), cm	97.5 (89.6-106.0)
Alcoholic drinks per week, median (IQR)	0 (0-1)
History of acute myocardial infarction	115 (11.7)
History of atrial fibrillation	56 (5.7)
Glomerular filtration rate, median (IQR), mL/min/1.73 m^2^	71.7 (56.8-88.3)
Prestroke systolic BP, mean (SD), mm Hg	140.4 (19.2)
Prestroke glucose, mean (SD), mg/dL	112.8 (41.2)
Prestroke LDL cholesterol, mean (SD), mg/dL	126.4 (34.2)
Antihypertensive medication use	671 (68.3)
Diabetes medication use	203 (20.9)
Cholesterol medication use	370 (37.9)
Prestroke cognitive scores, median (IQR)^c^	
General cognitive performance	52.7 (47.9-56.7)
Executive function	50.3 (44.0-55.2)
Memory	54.1 (50.1-56.6)
No. of prestroke cognitive assessments per individual, median (IQR)	
General cognitive performance	4 (2-7)
Executive function	2 (1-4)
Memory	3 (2-6)
Poststroke measures	
Follow-up time after stroke for primary outcome (global cognition), median (IQR), y	4.7 (2.6-7.9)
Systolic BP at first poststroke cognitive assessment, mean (SD), mm Hg	134.9 (21.9)
No. of systolic BP measurements after stroke, median (IQR)	1 (1-2)
Time from stroke to first poststroke systolic BP measurement, median (IQR), y	1.6 (0.7-4.1)
Glucose at first poststroke cognitive assessment, mean (SD), mg/dL	108.1 (34.4)
No. of glucose measurements after stroke, median (IQR)	1 (1-1)
Time from stroke to first poststroke glucose measurement, median (IQR), y	2.0 (0.9-4.0)
LDL cholesterol at first poststroke cognitive assessment, mean (SD), mg/dL	94.1 (34.9)
No. of LDL cholesterol measurements after stroke, median (IQR)	1 (0-1)
Time from stroke to first poststroke LDL cholesterol measurement, median (IQR), y	2.6 (1.2-5.0)
Cognitive scores at first poststroke cognitive assessment, median (IQR)	
General cognitive performance	49.1 (40.2-58.2)
Executive function	44.1 (37.5-52.5)
Memory	49.1 (43.5-57.4)
No. of poststroke cognitive assessments per individual, median (IQR)	
Global cognition	3 (1-4)
Executive function	2 (1-3)
Memory	2 (1-3)

Abbreviations: ARIC, Atherosclerosis Risk in Communities study; BP, blood pressure; CHS, Cardiovascular Health Study; FOS, Framingham Offspring Study; LDL, low-density lipoprotein; REGARDS, Reasons for Geographic And Racial Differences in Stroke study.

SI conversions: To convert glucose to millimoles per liter, multiply by 0.0555; LDL cholesterol to millimoles per liter, multiply by 0.0259.

^a^ Participants reporting race other than White or Black were excluded because REGARDS recruited White and Black participants by study design.

^b^ Body mass index is calculated as weight in kilograms divided by height in meters squared.

^c^ All cognitive measures are set to a *t* score metric (mean [SD], 50 [10]); a 1-point difference represents a 0.1-SD difference in the distribution of cognition across the 4 cohorts. Higher cognitive scores indicate better performance.

Models M1a, M1b, and M1c estimated the individual associations of poststroke SBP, glucose, and LDL cholesterol levels with cognitive decline separately by including the time-dependent cumulative mean of poststroke SBP (model M1a), glucose (model M1b), and LDL cholesterol (model M1c) level and their respective interactions with time. Model M2 estimated the combined association of poststroke SBP, glucose, and LDL cholesterol levels with cognitive decline by including the poststroke cumulative mean SBP, glucose, and LDL cholesterol levels and their interactions with time. A prespecified subgroup analysis was restricted to participants with APOE4 data and included the number of APOE4 alleles and its interaction with time. Statistical significance for all analyses was set as *P* < .05 (2-sided). All analyses were performed using SAS version 9.4 (SAS Institute). We calculated participant-specific predicted values for global cognition over 12 years for a 75-year-old female participant from the REGARDS cohort with typical values for all covariates using Stata version 16.1 (StataCorp).

To evaluate the robustness of our findings, we conducted 5 sensitivity analyses. We repeated analyses (1) treating poststroke cumulative mean SBP, glucose, and LDL cholesterol levels as time-invariant; 2) within the subgroup of participants with information on poststroke depressive symptoms before and after adjusting for poststroke depressive symptoms and poststroke depressive symptoms × time; (3) requiring participants to have at least 2 poststroke cognitive assessments (to assess attrition bias); (4) within individual cohorts (to assess heterogeneity across cohorts); and (5) including participants with baseline stroke history. The primary analyses used the observed glucose levels regardless of fasting status since the nonfasting glucose measurements still have information. A sensitivity analysis used estimated fasting glucose levels for nonfasting glucose measurements (eMethods in Supplement 1).

## Results

The study sample included 982 participants (480 [48.9%] were female individuals and 289 [29.4%] were Black individuals). Table 1 shows participant characteristics. Median (IQR) age at the time of stroke was 74.6 (69.1-79.8) years. During a median (IQR) follow-up of 4.7 (2.6-7.9) years, the median (IQR) number of poststroke cognitive assessments was 3 (1-4) for global cognition, 2 (1-3) for executive function, and 2 (1-3) for memory. eTable 1 in Supplement 1 shows participant characteristics by cohort. eTable 2 in Supplement 1 compares characteristics between included and excluded participants. The executive function analysis included 853 participants because the cognitive tests assessing this domain were administered less frequently. Of the 982 stroke survivors, 504 (51.3%) died during follow-up. Nevertheless, 781 (79.5%) had at least 2 poststroke cognitive assessments.

### Change in Global Cognition

Global cognition declined significantly over time after stroke (−0.46 points/y [95% CI, −0.65 to −0.27 points/y]; *P* < .001) (Table 2, model M1a). Examining each VRF separately, cumulative mean poststroke SBP, glucose, and LDL cholesterol levels were not associated with global cognitive decline (Table 2, models M1a, M1b, and M1c). Higher cumulative mean poststroke glucose level was associated with faster decline in global cognition (−0.04 points/y faster per 10–mg/dL increase [95% CI, −0.08 to −0.001 points/y]; *P* = .046) after accounting for cumulative mean poststroke SBP and LDL cholesterol levels (Table 2, model M2). Poststroke SBP and LDL cholesterol levels were not. The Figure shows global cognition slopes by cumulative mean poststroke glucose levels. Global cognition was 54.6 points at time of stroke for the exemplar. The range at 12 years after stroke was 42.9 (glucose level, 166 mg/dL) to 45.7 (glucose level, 86 mg/dL). The difference in global cognition between the highest and lowest glucose levels at 12 years after stroke was 2.8 points.

**Table 2.** Association of Poststroke Cumulative Mean Vascular Risk Factor Levels and Poststroke Global Cognition Decline^a^

Coefficient	Model M1a: time-varying cumulative mean poststroke systolic BP (n = 982)^b^		Model M1b: time-varying cumulative mean poststroke glucose (n = 787)^b^		Model M1c: time-varying cumulative mean poststroke LDL cholesterol (n = 734)^b^		Model M2: joint time-varying cumulative mean poststroke systolic BP, glucose, and LDL cholesterol (n = 609)^b^^,^^c^	
	Estimate (95% CI)	*P* value	Estimate (95% CI)	*P* value	Estimate (95% CI)	*P* value	Estimate (95% CI)	*P* value
Slope (change in cognition over time), per y	−0.46 (−0.65 to −0.27)	<.001	−0.35 (−0.52 to −0.18)	<.001	−0.41 (−0.59 to −0.22)	<.001	−0.52 (−0.75 to −0.30)	<.001
Changes to slope associated with								
Age at stroke (per 10-y increase), per y	−0.25 (−0.35 to −0.14)	<.001	−0.19 (−0.31 to −0.07)	.001	−0.21 (−0.32 to −0.10)	<.001	−0.20 (−0.32 to −0.08)	.001
Female sex, per y	−0.20 (−0.39 to −0.02)	.03	−0.29 (−0.50 to −0.08)	.007	−0.23 (−0.44 to −0.02)	.03	−0.29 (−0.51 to −0.06)	.01
Poststroke systolic BP (per 10–mm Hg increase), per y	0.03 (−0.01 to 0.08)	.16	NA	NA	NA	NA	0.04 (−0.03 to 0.11)	.22
Poststroke glucose (per 10–mg/dL increase), per y	NA	NA	−0.02 (−0.06 to 0.01)	.20	NA	NA	−0.04 (−0.08 to −0.001)	.046
Poststroke LDL cholesterol (per 10–mg/dL increase), per y	NA	NA	NA	NA	0.01 (−0.02 to 0.04)	.50	0.008 (−0.03 to 0.04)	.67

Abbreviations: BP, blood pressure; LDL, low-density lipoprotein; NA, not applicable.

^a^ All cognitive measures are set to a *t* score metric (mean [SD], 50 [10]); a 1-point difference represents a 0.1-SD difference in the distribution of cognition across the 4 cohorts. Higher cognitive scores indicate better performance. The median (IQR) number of global cognition assessments before and after stroke was 4 (2-7) and 3 (1-4), respectively.

^b^ Linear mixed-effects models included time since stroke, race, sex, age at time of stroke, cohort, education, income, medication for hypertension, diabetes, and high cholesterol, prestroke body mass index, waist circumference, smoking status, physical activity, alcohol consumption per week, history of myocardial infarction, history of atrial fibrillation, glomerular filtration rate, cohort study, prestroke mean global cognition, prestroke mean systolic BP, prestroke mean glucose, prestroke mean LDL cholesterol, poststroke mean systolic BP, poststroke mean glucose, poststroke mean LDL cholesterol, age at time of stroke × time since stroke, sex × time since stroke, poststroke mean systolic BP × time since stroke, poststroke mean glucose × time since stroke, poststroke mean LDL cholesterol × time since stroke, antihypertensive medication use × time, antihyperglycemic medication use × time, and lipid-lowering medication use × time. To consider correlation between longitudinal global cognition measures, random intercept and slope effect associated with participants were included. Glucose, LDL cholesterol, and systolic BP values are divided by 10 so that the parameter estimates refer to a 10-unit change in the variables. Each cognitive outcome is set to missing (censored) at the time of second expert-adjudicated incident stroke, death, loss to follow-up, or the end of follow-up, whichever occurs first. Models M1a, M1b, and M1c estimate the individual association of poststroke time-varying mean systolic BP, glucose, and LDL cholesterol levels with global cognitive decline with separate models. Model M1a includes a poststroke time-varying mean systolic BP level by time interaction and poststroke time-varying mean systolic BP. Model M1b includes a poststroke time-varying mean glucose level by time interaction and poststroke time-varying mean glucose. Model M1c includes poststroke time-varying mean LDL cholesterol level by time interaction and poststroke time-varying mean LDL cholesterol. The number of participants is smaller in models M1b, M1c, and M2 than model M1a because fewer participants had poststroke glucose and LDL cholesterol measures.

^c^ Model M2 estimates the joint association of poststroke time-varying mean systolic BP, glucose, and LDL cholesterol with poststroke global cognitive decline. Model M2 includes the poststroke time-varying mean systolic BP, glucose, and LDL cholesterol and their interactions with time.

**Figure.**
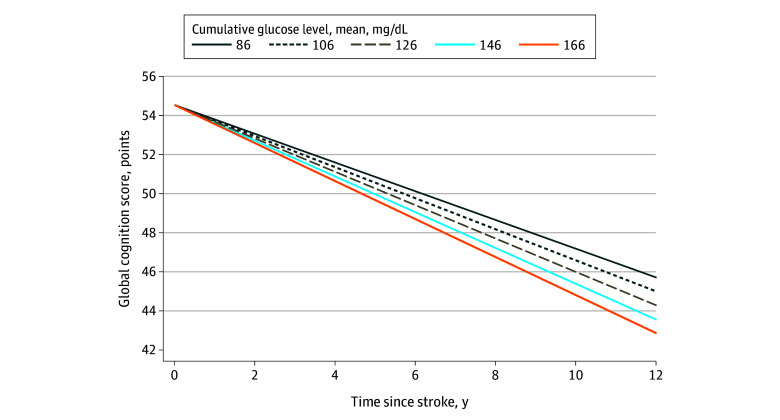
Conditional Predicted Values of Global Cognition Over Time by Cumulative Mean Glucose Levels To convert glucose to millimoles per liter, multiply by 0.0555.

There was no consistent evidence that history of diabetes at cohort baseline or diabetes present at time of first poststroke cognitive assessment significantly modified the association between poststroke glucose and cognitive decline (data not shown). Older age and female sex were associated with faster global cognitive decline (Table 2, all models).

### Changes in Executive Function and Memory

Executive function declined significantly over time after stroke (−0.47 points/y [95% CI, −0.68 to −0.26 points/y]; *P* < .001) (Table 3, model M1a). Poststroke glucose, LDL cholesterol, and SBP levels were not associated with executive function decline (Table 3, models M1a, M1b, M1c, and M2).

**Table 3.** Association of Poststroke Mean Vascular Risk Factor Levels and Poststroke Executive Function and Memory Decline

Coefficient	Model M1a: time-varying poststroke systolic BP^a^^,^^b^^,^^c^		Model M1b: time-varying poststroke glucose^a^^,^^b^^,^^c^		Model M1c: time-varying poststroke LDL cholesterol^a^^,^^b^^,^^c^		Model M2: Joint time-varying poststroke systolic BP, glucose, and LDL cholesterol^b^^,^^c^	
	Estimate (95% CI)	*P* value	Estimate (95% CI)	*P* value	Estimate (95% CI)	*P* value	Estimate (95% CI)	*P* value
**Executive function** ^d^ ^,^ ^e^								
Participants, total No.	853		667		609		495	
Slope (change in cognition over time), per y	−0.47 (−0.68 to −0.26)	<.001	−0.31 (−0.50 to −0.12)	.002	−0.35 (−0.55 to −0.15)	<.001	−0.50 (−0.76 to −0.24)	<.001
Changes to slope associated with								
Age at stroke (per 10-y increase), per y	−0.10 (−0.21 to 0.02)	.09	−0.02 (−0.15 to 0.11)	.78	−0.07 (−0.18 to 0.05)	.24	−0.07 (−0.20 to 0.06)	.28
Female sex, per y	−0.17 (−0.37 to 0.03)	.10	−0.21 (−0.45 to 0.02)	.07	−0.15 (−0.37 to 0.06)	.16	−0.22 (−0.47 to 0.02)	.07
Poststroke systolic BP (per 10–mm Hg increase), per y	−0.01 (−0.06 to 0.05)	.84	NA	NA	NA	NA	0.003 (−0.07 to 0.08)	.94
Poststroke glucose (per 10–mg/dL increase), per y	NA	NA	−0.003 (−0.05 to 0.04)	.90	NA	NA	−0.01 (−0.05 to 0.04)	.83
Poststroke LDL cholesterol (per 10–mg/dL increase), per y	NA	NA	NA	NA	−0.003 (−0.04 to 0.03)	.87	−0.01 (−0.05 to 0.03)	.57
**Memory** ^d^ ^,^ ^f^								
Participants, total No.	929		737		673		518	
Slope (change in cognition over time), per y	−0.22 (−0.42 to −0.01)	.04	−0.24 (−0.43 to −0.06)	.008	−0.24 (−0.45 to −0.03)	.03	−0.30 (−0.56 to −0.03)	.03
Changes to slope associated with								
Age at stroke (per 10-y increase), per y	0.02 (−0.10 to 0.14)	.73	0.02 (−0.10 to 0.16)	.72	−0.02 (−0.16 to 0.11)	.72	0.01 (−0.14 to 0.16)	.87
Female sex, per y	−0.29 (−0.50 to −0.07)	.01	−0.29 (−0.52 to −0.06)	.01	−0.33 (−0.57 to −0.08)	.009	−0.27 (−0.54 to −0.01)	.045
Poststroke systolic BP (per–10 mm Hg increase), per y	0.03 (−0.02 to 0.08)	.24	NA	NA	NA	NA	0.03 (−0.05 to 0.11)	.45
Poststroke glucose (per 10–mg/dL increase), per y	NA	NA	0.001 (−0.04 to 0.04)	.97	NA	NA	−0.01 (−0.06 to 0.04)	.69
Poststroke LDL cholesterol (per 10–mg/dL increase), per y	NA	NA	NA	NA	−0.003 (−0.04 to 0.03)	.87	0.01 (−0.03 to 0.05)	.80

Abbreviations: BP, blood pressure; LDL, low-density lipoprotein; NA, not applicable.

^a^ Linear mixed-effects models included time since stroke, race, sex, age at time of stroke, cohort, education, income, medication for hypertension, diabetes, and high cholesterol, prestroke body mass index, waist circumference, smoking status, physical activity, alcohol consumption per week, history of myocardial infarction, history of atrial fibrillation, glomerular filtration rate, cohort study, prestroke mean cognitive scores (executive function scores for executive function model and memory scores for memory model), prestroke mean systolic BP, prestroke mean glucose, prestroke mean LDL cholesterol, poststroke mean systolic BP, poststroke mean glucose, poststroke mean LDL cholesterol, age at time of stroke × time since stroke, sex × time since stroke, poststroke mean systolic BP × time since stroke, poststroke mean glucose × time since stroke, poststroke mean LDL cholesterol × time since stroke, antihypertensive medication use × time, antihyperglycemic medication use × time, and lipid-lowering medication use × time. To consider correlation between longitudinal cognition measures, random intercept and slope effect associated with participants were included. Glucose, LDL cholesterol, and systolic BP values are divided by 10 so that the parameter estimates refer to a 10-unit change in the variables. Each cognitive outcome is set to missing (censored) at the time of second expert-adjudicated incident stroke, death, loss to follow-up, or the end of follow-up, whichever occurs first.

^b^ Models M1a, M1b, and M1c estimate the individual association of poststroke time-varying mean systolic BP, glucose, and LDL cholesterol with cognitive decline scores (executive function or memory) with separate models. Model M1a includes a poststroke time-varying mean systolic BP level by time interaction and poststroke time-varying mean systolic BP. Model M1b includes a poststroke time-varying mean glucose level by time interaction and poststroke time-varying mean glucose. Model M1c includes poststroke time-varying mean LDL cholesterol level by time interaction and poststroke time-varying mean LDL cholesterol. Model M2 estimates the joint association of poststroke time-varying mean systolic BP, glucose, and LDL cholesterol on poststroke memory decline. Model M2 includes the poststroke time-varying mean systolic BP, glucose, and LDL cholesterol and their interactions with time.

^c^ The number of participants is smaller in models M1b, M1c, and M2 than model M1a because fewer participants had poststroke glucose and LDL cholesterol measures.

^d^ All cognitive measures are set to a *t* score metric (mean [SD], 50 [10]); a 1-point difference represents a 0.1-SD difference in the distribution of cognition across the 4 cohorts. Higher cognitive scores indicate better performance.

^e^ Median (IQR) number of executive function assessments before and after stroke was 2 (1-4) and 2 (1-3), respectively.

^f^ Median (25th, 75th interquartile range) number of memory assessments before stroke was 3 (2, 5) and after stroke was 2 (1, 3).

Memory declined significantly over time after stroke (−0.22 points/y [95% CI, −0.42 to −0.01 points/y]; *P* = .04) (Table 3, model M1a). Poststroke glucose, LDL cholesterol, and SBP levels were not associated with memory decline (Table 3, models M1a, M1b, M1c, and M2). Female sex was associated with faster memory decline (Table 3, models M1a, M1b, and M1c).

### Prespecified Subgroup Analysis Accounting for APOE4

We repeated models within the 798 participants with information on APOE4 before and after adjusting for APOE4 and APOE4 × time. A higher number of APOE4 alleles was associated with faster decline in global cognition and executive function after stroke (Table 4; eTables 3 and 4 in Supplement 1). Higher cumulative mean poststroke glucose level was associated with faster global cognition decline before and after adjusting for poststroke SBP and LDL cholesterol levels (before adjusting: −0.05 points/y faster per 10–mg/dL increase [95% CI, −0.09 to −0.01 points/y]; *P* = .01; after adjusting: −0.07 points/y faster per 10–mg/dL increase [95% CI, −0.11 to −0.03 points/y]; *P* = .002) (Table 4, models M1b and M2). There was no evidence of a significant APOE × glucose × time interaction for global cognition. No other associations between poststroke glucose, SBP, and LDL cholesterol levels with cognitive outcomes were found (Table 4; eTables 3 and 4 in Supplement 1).

**Table 4.** Prespecified Subgroup Analysis of Association of Poststroke Vascular Risk Factor Levels With Poststroke Global Cognition Decline Including Number of APOE4 Alleles Among Participants With APOE4 Information^a^

Coefficient	Model M1a: time-varying poststroke systolic BP (n = 798)^b^^,^^c^				Model M1b: Time-varying poststroke glucose (n = 627)^b^^,^^c^				Model M1c: Time-varying poststroke LDL cholesterol (n = 583)^b^^,^^c^				Model M2: Joint time-varying poststroke systolic BP, glucose, and LDL cholesterol (n = 472)^c^			
	Without APOE4		With APOE4		Without APOE4		With APOE4		Without APOE4		With APOE4		Without APOE4		With APOE4	
	Estimate (95% CI)	*P* value	Estimate (95% CI)	*P* value	Estimate (95% CI)	*P* value	Estimate (95% CI)	*P* value	Estimate (95% CI)	*P* value	Estimate (95% CI)	*P* value	Estimate (95% CI)	*P* value	Estimate (95% CI)	*P* value
Slope (change in cognition over time), per y	−0.46 (−0.67 to −0.26)	<.001	−0.34 (−0.55 to −0.13)	.002	−0.34 (−0.52 to −0.16)	<.001	−0.17 (−0.36 to 0.02)	.08	−0.41 (−0.62 to −0.20)	<.001	−0.35 (−0.56 to −0.15)	<.001	−0.54 (−0.81 to −0.28)	<.001	−0.41 (−0.66 to −0.16)	.001
Changes to slope associated with																
Age at stroke (per 10-y increase), per y	−0.26 (−0.37 to −0.14)	<.001	−0.28 (−0.39 to −0.17)	<.001	−0.20 (−0.33 to −0.08)	.001	−0.24 (−0.36 to −0.12)	<.001	−0.21 (−0.33 to −0.09)	<.001	−0.24 (−0.36 to −0.13)	<.001	−0.21 (−0.34 to −0.07)	.002	−0.25 (−0.37 to −0.13)	<.001
Female sex, per y	−0.23 (−0.43 to −0.03)	.02	−0.23 (−0.42 to −0.03)	.02	−0.33 (−0.56 to −0.11)	.004	-.0.36 (−0.57 to −0.14)	.001	−0.26 (−0.48 to −0.03)	.03	−0.23 (−0.44 to −0.01)	.04	−0.31 (−0.56 to −0.06)	.01	−0.32 (−0.54 to −0.08)	.007
Poststroke systolic BP (per 10–mm Hg increase), per y	0.03 (−0.02 to 0.09)	.23	0.04 (−0.02 to 0.09)	.19	NA	NA	NA	NA	NA	NA	NA	NA	0.02 (−0.06 to 0.09)	.64	0.02 (−0.05 to 0.09)	.52
Poststroke glucose (per 10–mg/dL increase), per y	NA	NA	NA	NA	−0.04 (−0.08 to 0.00)	.07	−0.05 (−0.09 to −0.01)	.01	NA	NA	NA	NA	−0.05 (−0.10 to −0.00)	.03	−0.07 (−0.11 to −0.03)	.002
Poststroke LDL cholesterol (per 10–mg/dL increase), per y	NA	NA	NA	NA	NA	NA	NA	NA	0.01 (−0.03 to 0.04)	.68	0.02 (−0.02 to 0.05)	.33	0.003 (−0.04 to 0.04)	.86	0.02 (−0.02 to 0.05)	.37
1 APOE4 allele vs 0 APOE4 alleles, per y	NA	NA	−0.31 (−0.53 to −0.09)	.007	NA	NA	−0.38 (−0.62 to −0.14)	.002	NA	NA	−0.30 (−0.55 to −0.06)	.02	NA	NA	−0.34 (−0.59 to −0.08)	.009
2 APOE4 alleles vs 0 APOE4 alleles, per year	NA	NA	−0.70 (−1.40 to 0.003)	.05	NA	NA	−1.02 (−1.8 to −0.27)	.008	NA	NA	−1.00 (−1.74 to −0.25)	.009	NA	NA	−1.43 (−2.20 to −0.66)	<.001

Abbreviations: APOE4, apolipoprotein E4; BP, blood pressure; LDL, low-density lipoprotein; NA, not applicable.

^a^ Cognitive measures are set to a *t* score metric (mean [SD], 50 [10]); a 1-point difference represents a 0.1-SD difference in the distribution of cognition across the 4 cohorts. Higher cognitive scores indicate better performance. Median (IQR) number of global cognition assessments before and after stroke was 3 (2-6) and 2 (1-4), respectively.

^b^ Linear mixed-effects models included number of APOE4 alleles, time since stroke, race, sex, age at time of stroke, cohort, education, income, medication for hypertension, diabetes, and high cholesterol, prestroke body mass index, waist circumference, smoking status, physical activity, alcohol consumption per week, history of myocardial infarction, history of atrial fibrillation, glomerular filtration rate, cohort study, prestroke mean global cognition, prestroke mean systolic BP, prestroke mean glucose, prestroke mean LDL cholesterol, poststroke mean systolic BP, poststroke mean glucose, poststroke mean LDL cholesterol, age at time of stroke × time since stroke, sex × time since stroke, poststroke mean systolic BP × time since stroke, poststroke mean glucose × time since stroke, poststroke mean LDL cholesterol × time since stroke, antihypertensive medication use × time, antihyperglycemic medication use × time, and lipid-lowering medication use × time. To consider correlation between longitudinal global cognition measures, random intercept and slope effect associated with participants were included. Glucose, LDL cholesterol, and systolic BP values are divided by 10 so that the parameter estimates refer to a 10-unit change in the variables. Each cognitive outcome is set to missing (censored) at the time of second expert-adjudicated incident stroke, death, loss to follow-up, or the end of follow-up, whichever occurs first.

^c^ Models M1a, M1b, and M1c estimate the individual association of poststroke time-varying mean systolic BP, glucose, and LDL cholesterol with poststroke executive function decline with separate models, and model M2 estimates the joint association of these measures. See the Statistical Analysis section for more details. The number of participants is smaller in models M1b, M1c, and M2 than model M1a because fewer participants have poststroke glucose and LDL measures.

### Sensitivity Analyses

Higher time-invariant cumulative mean poststroke glucose level was associated with faster decline in global cognition, but not executive function or memory, before and after accounting for time-invariant cumulative mean poststroke SBP and LDL cholesterol levels (before: −0.05 points/y faster per 10–mg/dL increase [95% CI, −0.07 to −0.02]; *P* < .001; after: −0.06 points/y faster per 10–mg/dL increase [95% CI, −0.09 to −0.03 points/y]; *P* < .001) (eTable 5 in Supplement 1, models M1b and M2, and eTables 6 and 7 in Supplement 1, model M2). Poststroke mean LDL cholesterol and SBP levels were not associated with cognitive outcomes (eTables 5, 6, and 7 in Supplement 1, model M2). Results were consistent in analyses including participants with baseline stroke history (eTables 8, 9, and 10 in Supplement 1), requiring at least 2 poststroke cognitive assessments (eTable 11 in Supplement 1), and accounting for poststroke depressive symptoms, although some contrasts were no longer significant (eTables 12, 13, and 14 in Supplement 1). Results were consistent across 3 of 4 cohorts (eTables 15, 16, and 17 in Supplement 1). Among 982 participants, 960 participants (97.8%) had 1 or more fasting glucose measurements (236 ARIC, 330 CHS, 101 FOS, and 293 REGARDS), and 22 participants (2.2%) had nonfasting glucose measurements only (2 ARIC, 2 CHS, 0 FOS, and 18 REGARDS). Results were consistent with estimated fasting glucose for nonfasting glucose measurements (eTables 18, 19, and 20 in Supplement 1).

## Discussion

In this cohort study with an IPD meta-analysis of 982 stroke survivors from 4 prospective cohort studies, higher cumulative mean poststroke glucose levels were associated with faster decline in global cognition, but not executive function or memory, after accounting for poststroke SBP and LDL cholesterol levels. In the prespecified subgroup of 798 individuals with data on APOE4, higher cumulative mean poststroke glucose level was associated with a faster global cognitive decline before and after adjusting for poststroke SBP and LDL cholesterol levels. We found no evidence that poststroke SBP and LDL cholesterol levels were associated with cognitive decline.

Some studies have shown that prevalent diabetes at the time of stroke is associated with greater risk for poststroke dementia and cognitive decline.^2,33,34,35,36^ Our results extend prior research by providing evidence that higher cumulative mean glucose level after stroke hospitalization is independently associated with faster global cognitive decline, controlling for prestroke objective levels of glucose, BP, LDL cholesterol, and cognition, regardless of diabetes status. Our results suggest that accounting for confounding variables (poststroke BP and LDL cholesterol levels in the primary analysis and APOE4 in the prespecified subgroup analysis) was necessary to detect the association between poststroke hyperglycemia and accelerated global cognitive decline. We found no evidence that APOE4 modified the effect of poststroke glucose level on global cognitive decline.

Our results are consistent with a study suggesting higher blood glucose is a dementia risk factor even in adults without diabetes.^9^ Poststroke hyperglycemia might accelerate cognitive decline through cerebral microvascular injury, oxidative stress, inflammation, and neurodegeneration.^37,38,39,40,41^ Although APOE4 might amplify diabetes’s effect on Alzheimer disease (AD),^42^ APOE4 did not modify the association between poststroke glucose and global cognitive decline, suggesting cerebrovascular and non-AD neurodegenerative pathways might underlie glucose-associated cognitive decline in stroke survivors.^41^ It is unlikely that high BP and clinically apparent recurrent strokes explain the observed glucose-associated poststroke cognitive decline because models controlled for prestroke and poststroke BP levels and censored cognitive observations at the time of recurrent stroke. Nevertheless, stroke survivors with high glucose levels could have had subclinical infarcts after their index stroke that contributed to cognitive decline. Brain imaging data after the incident stroke was unavailable. These findings suggest a scientific need to determine the mechanisms of glucose-associated poststroke cognitive decline.

Contrary to our hypothesis, we found no evidence of an association between either poststroke SBP or LDL cholesterol levels and cognitive decline. One explanation is that they are not associated. Consistent with our results, a study showed that diabetes, but not hypertension or hyperlipidemia, was associated with poststroke dementia.^2^ While some observational studies have found that elevated prestroke SBP and LDL cholesterol levels are associated with a higher poststroke dementia risk,^33,43,44,45^ they have not reported greater risk for poststroke hypertension and hypercholesterolemia consistently.^46^ BP-lowering trials to prevent dementia have excluded patients with stroke and diabetes, and trials of intensive BP and lipid lowering to prevent poststroke dementia and cognitive decline, independent of recurrent stroke, have been negative.^47,48,49,50,51,52^ Another explanation is the executive function and memory measures might not have detected the poststroke glucose–cognitive decline association. Alternatively, the sample’s older age might explain the null finding. Evidence that high BP and LDL cholesterol levels are associated with cognitive decline in stroke-free adults is strong in midlife but unclear in older age, whereas diabetes is associated with cognitive decline at both ages.

The finding that older age and APOE4 are associated with faster poststroke cognitive decline is consistent with prior evidence.^33,53^ Sex differences in poststroke dementia risk are unclear, with a meta-analysis finding greater risk for female participants but significant heterogeneity across studies.^33^ We provide evidence that female sex is associated with faster poststroke cognitive decline.

Study strengths are the large sample size, inclusion of many Black stroke survivors, and population-based sampling, which increases generalizability and is unique for a longitudinal design. The objective measurements of VRFs and cognition before and after incident stroke enabled us to estimate the associations of time-dependent cumulative mean poststroke VRF levels with cognitive decline, controlling for mean prestroke VRF and cognition levels. Each cohort systematically measured cognitive domains affected by VRFs: global cognition, memory, and executive function.^18,36^ Results were robust to sensitivity analyses and consistent across 3 of 4 cohorts.

### Limitations

This study has limitations. Information on stroke severity,^2^ stroke type,^28^ premorbid and poststroke functional status,^2^ leukoaraiosis,^2^ dysphasia,^2^ brain atrophy,^4,33^ stroke location,^2,54^ and traumatic brain injury after stroke was not available for the analysis, although stroke severity did not explain diabetes’ higher risk of poststroke dementia.^2^ The linear-effects models do not allow accounting for competing risks of death, and selective attrition of cognitively impaired stroke survivors could underestimate the rate of cognitive decline.^55^ However, a sensitivity analysis requiring at least 2 poststroke cognitive assessments had similar results. We did not study incident dementia or cognitive impairment because 1 cohort (REGARDS) lacked this information at the time of this study. Our assumption that participants’ postmortem cognitive data are missing at random might lead to immortal cohort bias and underestimate cognitive declines. However, it is valid to answer the research question quantifying differences in cognitive trajectories associated with different poststroke VRF levels through study follow-up. Although using a fixed effect for cohorts might produce conservative estimates of differences in cognitive slopes, we also performed within-cohort analyses and results were consistent with findings from the pooled analysis. We could not examine other cognitive domains (eg, language, visuoperception). Young stroke survivors with higher prestroke cognitive scores were more likely to be excluded, increasing our ability to detect cognitive decline. Diabetes medication use might be lower than previously reported because many participants only had prestroke medication data available and as many as 20% of patients with stroke might have undiagnosed diabetes.^56^

If causal, the association of cumulative mean poststroke glucose levels with global cognitive decline may have a small clinical effect in absolute terms, but it is within an order of magnitude similar to that of aging (the ratio of the slope coefficients indicates 1.4 to 2.9 years of aging per 10–mg/dL higher glucose level) and the population-level impact would be significant. The prevalence of hyperglycemia in stroke survivors has increased steadily since 2004.^11^ Clinical guidelines recommend individualized glycemic control in stroke survivors to prevent microvascular complications because the optimal levels of glucose to prevent recurrent stroke are unknown.^57^ Findings suggest that stroke survivors with hyperglycemia warrant close monitoring for cognitive impairment. Research assessing whether effective interventions to improve glycemic control in stroke survivors are potential strategies to reduce cognitive decline is needed.

## Conclusions

In this study with an IPD meta-analysis of 4 cohort studies, higher poststroke glucose levels were associated with faster global cognitive decline. We found no evidence that poststroke SBP and LDL cholesterol levels were associated with cognitive decline.

## Notes

Supplement 1.
eMethods.
**eFigure.** Derivation of the Participant Cohort **eTable 1.** Characteristics of Participants at First Poststroke Cognitive Assessment in the Pooled Cohort Sample by Cohort: STROKE COG Study, 1971 to 2021 **eTable 2.** Descriptive Comparison of Included (n=982) vs Excluded (n=138) Participants **eTable 3.** Sensitivity Analysis of Association of Poststroke Vascular Risk Factor Levels With Poststroke Executive Function Decline Including Number of APOE4 Alleles Among Participants With APOE4 Information: STROKE COG Study, 1971 to 2021 **eTable 4.** Sensitivity Analysis of Association of Poststroke Vascular Risk Factor Levels With Poststroke Memory Decline Including Number of APOE4 Alleles Among Participants With APOE4 Information: STROKE COG Study, 1971 to 2019 **eTable 5.** Sensitivity Analysis of Association of Poststroke Time-Invariant Mean Vascular Risk Factor Levels and Poststroke Global Cognition Decline: STROKE COG Study, 1971 to 2021 **eTable 6.** Association of Poststroke Invariant Mean Vascular Risk Factor Levels and Poststroke Executive Function Decline: STROKE COG Study, 1971 to 2021 **eTable 7.** Association of Poststroke Time-Invariant Mean Vascular Risk Factor Levels and Poststroke Memory Decline: STROKE COG Study, 1971 to 2021 **eTable 8.** Sensitivity Analysis of Association of Poststroke Vascular Risk Factor Levels and Poststroke Global Cognition Decline Including Participants With History of Stroke at Cohort Baseline: STROKE COG Study, 1971 to 2021 **eTable 9.** Association of Poststroke Invariant Mean Vascular Risk Factor Levels and Poststroke Executive Function Decline Including Participants With History of Stroke at Cohort Baseline: STROKE COG Study, 1971 to 2021 **eTable 10.** Association of Poststroke Time-Invariant Mean Vascular Risk Factor Levels and Poststroke Memory Decline Including Participants With History of Stroke at Cohort Baseline: STROKE COG Study, 1971 to 2021 **eTable 11.** Sensitivity Analysis of Association of Poststroke Vascular Risk Factor Levels and Post-Stroke Global Cognition Decline Requiring Participants to Have 2 or More Poststroke Cognitive Assessments: STROKE COG Study, 1971 to 2021 **eTable 12.** Sensitivity Analysis of Association of Poststroke Vascular Risk Factor Levels With Poststroke Global Cognition Decline Including Poststroke Depressive Symptoms Among Participants With Depressive Symptom Scores: STROKE COG Study, 1971 to 2021 **eTable 13.** Sensitivity Analysis of Association of Poststroke Vascular Risk Factor Levels With Poststroke Executive Function Decline Including Poststroke Depressive Symptoms Among Participants With Depressive Symptom Scores: STROKE COG Study, 1971 to 2021 **eTable 14.** Sensitivity Analysis of Association of Poststroke Vascular Risk Factor Levels With Poststroke Memory Decline Including Poststroke Depressive Symptoms Among Participants With Depressive Symptom Scores: STROKE COG Study, 1971 to 2019 **eTable 15.** Sensitivity Analysis of Association of Poststroke Vascular Risk Factor Levels With Poststroke Global Cognition Decline by Cohort: STROKE COG Study, 1971 to 2021 **eTable 16.** Sensitivity Analysis of Association of Poststroke Vascular Risk Factor Levels With Post-Stroke Executive Function Decline by Cohort: STROKE COG Study, 1971 to 2019 **eTable 17.** Sensitivity Analysis of Association of Poststroke Vascular Risk Factor Levels With Poststroke Memory Decline by Cohort: STROKE COG Study, 1971 to 2019 **eTable 18.** Sensitivity Analysis of Association of Post-Stroke Vascular Risk Factor Levels with Post-Stroke Global Cognition Decline with Estimated Fasting Glucose Levels: STROKE COG Study, 1971 to 2021 **eTable 19.** Sensitivity Analysis of Association of Post-Stroke Vascular Risk Factor Levels with Post-Stroke Executive Function Decline with Estimated Fasting Glucose Levels: STROKE COG Study, 1971 to 2021 **eTable 20.** Sensitivity Analysis of Association of Post-Stroke Vascular Risk Factor Levels with Post-Stroke Memory Decline with Estimated Fasting Glucose Levels: STROKE COG Study, 1971 to 2021
eReferences.

Supplement 2.
Data Sharing Statement
