# Errors in Methods and Results Sections, Figure, Table 1, and Supplement 1

**DOI:** 10.1001/jamanetworkopen.2024.11800

**Published:** 2024-04-11

In the Original Investigation “Associations Between Vascular Risk Factor Levels and Cognitive Decline Among Stroke Survivors,”^1^ published May 17, 2023, there were errors in the Methods and Results sections, the Figure, and Table 1. These sections should have stated that all observed glucose levels, rather than fasting glucose levels, were used. Additionally, 3 eTables were added to Supplement 1 with results from a sensitivity analysis using estimated fasting glucose levels. The authors have explained what happened in a Comment.^2^
