# Supplementary material for: Associations Between Vascular Risk Factor Levels and Cognitive Decline Among Stroke Survivors
Source: JAMA Netw Open. 2023 May 17;6(5):e2313879. doi: 10.1001/jamanetworkopen.2023.13879 (PMC10193182; doi:10.1001/jamanetworkopen.2023.13879)
Supplement: Supplement 1. — eMethods. eFigure. Derivation of the Participant Cohort eTable 1. Characteristics of Participants at First Poststroke Cognitive Assessment in the Pooled Cohort Sample by Cohort: STROKE COG Study, 1971 to 2021 eTable 2. Descriptive Comparison of Included (n=982) vs Excluded (n=138) Participants eTable 3. Sensitivity Analysis of Association of Poststroke Vascular Risk Factor Levels With Poststroke Executive Function Decline Including Number of APOE4 Alleles Among Participants With APOE4 Information: STROKE COG Study, 1971 to 2021 eTable 4. Sensitivity Analysis of Association of Poststroke Vascular Risk Factor Levels With Poststroke Memory Decline Including Number of APOE4 Alleles Among Participants With APOE4 Information: STROKE COG Study, 1971 to 2019 eTable 5. Sensitivity Analysis of Association of Poststroke Time-Invariant Mean Vascular Risk Factor Levels and Poststroke Global Cognition Decline: STROKE COG Study, 1971 to 2021 eTable 6. Association of Poststroke Invariant Mean Vascular Risk Factor Levels and Poststroke Executive Function Decline: STROKE COG Study, 1971 to 2021 eTable 7. Association of Poststroke Time-Invariant Mean Vascular Risk Factor Levels and Poststroke Memory Decline: STROKE COG Study, 1971 to 2021 eTable 8. Sensitivity Analysis of Association of Poststroke Vascular Risk Factor Levels and Poststroke Global Cognition Decline Including Participants With History of Stroke at Cohort Baseline: STROKE COG Study, 1971 to 2021 eTable 9. Association of Poststroke Invariant Mean Vascular Risk Factor Levels and Poststroke Executive Function Decline Including Participants With History of Stroke at Cohort Baseline: STROKE COG Study, 1971 to 2021 eTable 10. Association of Poststroke Time-Invariant Mean Vascular Risk Factor Levels and Poststroke Memory Decline Including Participants With History of Stroke at Cohort Baseline: STROKE COG Study, 1971 to 2021 eTable 11. Sensitivity Analysis of Association of Poststroke Vascular Risk Factor Levels and Post-Stroke [file jamanetwopen-e2313879-s001.pdf]

Supplemental Online Content

Levine DA, Chen B, Galecki AT, et al. Associations between vascular risk factor levels and cognitive decline among stroke survivors. *JAMA Netw Open.* 2023;6(5):e2313879. doi:10.1001/jamanetworkopen.2023.13879

eMethods.

eFigure. Derivation of the Participant Cohort

eTable 1. Characteristics of Participants at First Poststroke Cognitive Assessment in the Pooled Cohort Sample by Cohort: STROKE COG Study, 1971 to 2021

eTable 2. Descriptive Comparison of Included (n=982) vs Excluded (n=138) Participants

eTable 3. Sensitivity Analysis of Association of Poststroke Vascular Risk Factor Levels With Poststroke Executive Function Decline Including Number of APOE4 Alleles Among Participants With APOE4 Information: STROKE COG Study, 1971 to 2021

eTable 4. Sensitivity Analysis of Association of Poststroke Vascular Risk Factor Levels With Poststroke Memory Decline Including Number of APOE4 Alleles Among Participants With APOE4 Information: STROKE COG Study, 1971 to 2019

eTable 5. Sensitivity Analysis of Association of Poststroke Time-Invariant Mean Vascular Risk Factor Levels and Poststroke Global Cognition Decline: STROKE COG Study, 1971 to 2021

eTable 6. Association of Poststroke Invariant Mean Vascular Risk Factor Levels and Poststroke Executive Function Decline: STROKE COG Study, 1971 to 2021

eTable 7. Association of Poststroke Time-Invariant Mean Vascular Risk Factor Levels and Poststroke Memory Decline: STROKE COG Study, 1971 to 2021

eTable 8. Sensitivity Analysis of Association of Poststroke Vascular Risk Factor Levels and Poststroke Global Cognition Decline Including Participants With History of Stroke at Cohort Baseline: STROKE COG Study, 1971 to 2021

eTable 9. Association of Poststroke Invariant Mean Vascular Risk Factor Levels and Poststroke Executive Function Decline Including Participants With History of Stroke at Cohort Baseline: STROKE COG Study, 1971 to 2021

eTable 10. Association of Poststroke Time-Invariant Mean Vascular Risk Factor Levels and Poststroke Memory Decline Including Participants With History of Stroke at Cohort Baseline: STROKE COG Study, 1971 to 2021

eTable 11. Sensitivity Analysis of Association of Poststroke Vascular Risk Factor Levels and Post-Stroke Global Cognition Decline Requiring Participants to Have 2 or More Poststroke Cognitive Assessments: STROKE COG Study, 1971 to 2021

eTable 12. Sensitivity Analysis of Association of Poststroke Vascular Risk Factor Levels With Poststroke Global Cognition Decline Including Poststroke Depressive Symptoms Among Participants With Depressive Symptom Scores: STROKE COG Study, 1971 to 2021

© 2023 Levine DA et al. *JAMA Network Open.*

eTable 13. Sensitivity Analysis of Association of Poststroke Vascular Risk Factor Levels With Poststroke Executive Function Decline Including Poststroke Depressive Symptoms Among Participants With Depressive Symptom Scores: STROKE COG Study, 1971 to 2021

eTable 14. Sensitivity Analysis of Association of Poststroke Vascular Risk Factor Levels With Poststroke Memory Decline Including Poststroke Depressive Symptoms Among Participants With Depressive Symptom Scores: STROKE COG Study, 1971 to 2019

eTable 15. Sensitivity Analysis of Association of Poststroke Vascular Risk Factor Levels With Poststroke Global Cognition Decline by Cohort: STROKE COG Study, 1971 to 2021

eTable 16. Sensitivity Analysis of Association of Poststroke Vascular Risk Factor Levels With Post-Stroke Executive Function Decline by Cohort: STROKE COG Study, 1971 to 2019

eTable 17. Sensitivity Analysis of Association of Poststroke Vascular Risk Factor Levels With Poststroke Memory Decline by Cohort: STROKE COG Study, 1971 to 2019

eTable 18. Sensitivity Analysis of Association of Post-Stroke Vascular Risk Factor Levels with Post-Stroke Global Cognition Decline with Estimated Fasting Glucose Levels: STROKE COG Study, 1971 to 2021

eTable 19. Sensitivity Analysis of Association of Post-Stroke Vascular Risk Factor Levels with Post-Stroke Executive Function Decline with Estimated Fasting Glucose Levels: STROKE COG Study, 1971 to 2021

eTable 20. Sensitivity Analysis of Association of Post-Stroke Vascular Risk Factor Levels with Post-Stroke Memory Decline with Estimated Fasting Glucose Levels: STROKE COG Study, 1971 to 2021

eReferences.

This supplemental material has been provided by the authors to give readers additional information about their work.

© 2023 Levine DA et al. *JAMA Network Open.*

eMethods.

Cohort Studies

The Atherosclerosis Risk in Communities Study (ARIC) (https://www2.cscc.unc.edu/aric/desc) is a study of the causes of atherosclerosis and its clinical outcomes and variation in cardiovascular disease (CVD) risk factors, medical care, and disease by race, gender, location, and date. From 1987-1989, the study recruited 15,792 adults aged 45-64 (28% Black, 72% White) from 4 US communities (Forsyth County, NC; Jackson, MS; Minneapolis, MN; and Washington County, MD). Participants have had follow-up exams every 2-13 years for 25+ years. Starting at the 2nd exam, every exam measured cognition (n=14,348) using a short battery. We did not use data from the 3rd exam when only a very small proportion of the cohort had cognition assessed. ARIC has a neurocognitive sub-study to perform dementia surveillance. ARIC expanded the neuropsychology battery in exams 5-7. We included the neurocognitive sub-study and expanded neuropsychology battery in the analysis.

The Cardiovascular Health Study (CHS) (http://www.chs-nhlbi.org/) is a study of risk factors for CVD in older adults. In 1989-1990, the study recruited 5,201 adults aged ≥65 (4.7% Black, 95.3% White) from 4 US communities (Sacramento County, CA; Washington County, MD; Forsyth County, NC; and Pittsburgh, PA). In 1992-1993, the study added a supplementary cohort of 687 Blacks to 3 of the 4 US communities listed above (the Washington County, MD community was excluded from this cohort), bringing the total race distribution to 16% Black and 84% White. We used in-person cognitive test data from study years 3-11 and TICS data from years 8-11 and 20-25. Data on test timing (days from baseline) were not available after year 25. CHS also had a dementia sub-study that added a neuropsychology battery and neurology exam, which we included in the analysis.

© 2023 Levine DA et al. *JAMA Network Open.*

The Framingham Offspring Study (FOS) (https://www.framinghamheartstudy.org/index.php) is a study of risk factors for CVD. In 1971, the study recruited 5,124 adults aged <10-70 (>95% White) from New England. Follow-up exams have occurred every 4-8 years for 45 years. All exams from exam 5 forward measured cognition using Mini-Mental State Examination (MMSE). In 1999-2000, as a call-back after exam 7, FOS began a cognitive and MRI sub-study that added an expanded neuropsychology battery. The same battery was administered again after exam 8. We included the expanded neuropsychology battery from both exams in the analysis. We included FOS because it contributes individual participant data to estimate post-stroke cognitive decline in White individuals as well as the effect of time-dependent post-stroke cumulative mean systolic blood pressure (SBP), glucose and low-density lipoprotein (LDL) cholesterol levels on cognitive decline.

The Reasons for Geographic and Racial Differences in Stroke (REGARDS) (https://www.uab.edu/soph/regardsstudy/) is a study of the regional and racial influences on stroke mortality in Blacks and Whites. From 2003-2007, the study recruited 30,239 adults aged ≥45 (42% Blacks, 55% women) from the continental United States (US), with oversampling of the Southeastern US. In-home examinations collected vascular risk factors at baseline and ~10 years later. REGARDS assesses cognition longitudinally by telephone using the Six-Item Screener beginning in 2003 and measured annually, and an expanded neuropsychology battery measured bi-annually starting in 2006.

Harmonization of Cognitive Function Assessments

In a pre-statistical harmonization phase, we identified 109 test items from 27 cognitive instruments across the cohorts and determined shared items between cohorts following previous methods.[1,2] Expert neuropsychologists (EMB, BJG) assigned each test item to a cognitive domain. In Item Response Theory (IRT), each test item is weighted based on its

© 2023 Levine DA et al. *JAMA Network Open.*

correlation with other items and empirically assigned a relative location along the latent trait (e.g., global cognition) corresponding to its estimated difficulty. All available cognitive test items, including tests that are common across cohorts and tests that are unique to individual cohorts, were used. We computed factor scores from models for each domain using the regression-based method in Mplus version 8.2.[3,4] We used equipercentile equating to adjust the distributions of some like test items (e.g., animal recall and Block Design) across cohorts to be on a common scale prior to IRT co-calibration.[5] This IRT approach produces cognitive scores that are more precise than those derived from a z-score approach that standardizes and averages test scores.[6] The measurement model for each cognitive factor was constructed such that cognitive tests provided no information about cognitive levels at study exams in which the tests were not administered. Although the cognitive factors were constructed in this way, there remains an autocorrelation across time within people that is modeled using random effects models described in the main analysis section.

Cognitive Tests by Domain

Global cognition domain: Animal Naming (ARIC, CHS, FOS, REGARDS), Baddeley and Papagno Divided Attention Task (CHS), Block Design Test (FOS: WAIS; CHS: WAIS-R), Boston Naming Test (ARIC, CHS, FOS), California Verbal Learning Test (CHS), Delayed Word Recall test (ARIC), Consortium to Establish a Registry for Alzheimer's Disease (CERAD) Word List Learning (REGARDS), Digit Span Test (ARIC: WMS-R; CHS: WAIS-R; FOS: WAIS), Digit Symbol Substitution Test/Digit Symbol Coding Test (ARIC: WAIS-R), Finger Tapping Test (FOS, ARIC), Grooved Pegboard Test (CHS), Hooper Visual Organization Test (FOS), Letter Fluency Test (ARIC, CHS, FOS, REGARDS), Logical Memory Test (ARIC: WMS-R; FOS: WMS), Mini-Mental State Examination (ARIC, CHS, FOS), Modified Mini-Mental State Examination (CHS), Montreal Cognitive Assessment (REGARDS), Paired Associates Learning (WMS; FOS), Ravens Colored Progressive Matrices (CHS), Rey-Osterrieth Complex Figure

© 2023 Levine DA et al. *JAMA Network Open.*

Test (CHS), Semantic Word Generation (CHS), Similarities (WAIS; FOS), Six Item Screener (REGARDS), Stroop Neuropsychological Screening Test (CHS), Telephone Interview for Cognitive Status (ARIC, CHS), Trail Making Test (ARIC, CHS, FOS), Visual Reproduction (WMS; FOS).

Memory domain: California Verbal Learning Test (CHS), Consortium to Establish a Registry for Alzheimer's Disease (CERAD) Word List Learning (REGARDS), Delayed Word Recall test (ARIC), Digit Symbol Substitution Test (Incidental Learning; ARIC), Logical Memory Test (ARIC: WMS-R; FOS: WMS), Mini-Mental State Examination recall items (ARIC), Modified Mini-Mental State Examination delayed recall items (CHS); Telephone Interview for Cognitive Status recall items (ARIC, CHS); Montreal Cognitive Assessment recall items (REGARDS), Paired Associates Learning (WMS; FOS), Rey Complex Figure Test Recall (CHS); Visual Reproduction (WMS; FOS).

Executive function domain: Animal Naming (ARIC, CHS, FOS, REGARDS), Baddeley and Papagno Divided Attention Task (CHS), Block Design Test (FOS: WAIS; CHS: WAIS-R); California Verbal Learning Test (CHS; semantic clustering), Digit Span Test (ARIC: WMS-R; CHS: WAIS-R; FOS: WAIS), Digit Symbol Substitution Test (ARIC, CHS: WAIS-R), Letter Fluency Test (ARIC, CHS, FOS, REGARDS), Mini-Mental State Examination subtraction/backward spelling items (ARIC; CHS; FOS); Modified Mini-Mental State Examination) animal naming, counting, similarities items (ARIC); Ravens Colored Progressive Matrices (CHS); Rey-Osterrieth Complex Figure Test Copy (CHS); Semantic Word Generation (CHS), Similarities (WAIS; FOS), Stroop Neuropsychological Screening Test (CHS), Telephone Interview for Cognitive Status items counting and subtraction items (ARIC, CHS); Trail Making Test (ARIC, CHS, FOS).

© 2023 Levine DA et al. *JAMA Network Open.*

Note: WAIS is Wechsler Adult Intelligence Scale. WAIS-R is Wechsler Adult Intelligence Scale – Revised. WAIS-III is Wechsler Adult Intelligence Scale – 3rd Edition. WMS is Wechsler Memory Scale. WMS-R is Wechsler Memory Scale – Revised. WMS-III is Wechsler Memory Scale – 3rd Edition.

Cognitive tests were administered based on the protocols and schedules of the cohort studies and their ancillary studies. Information on the timing of the administration of each cognitive test by cohort is available on request. In three cohorts (ARIC, CHS, FOS), trained staff administered cognitive tests by telephone for participants unable to attend some exams in-person.

Measurement of Post-stroke Vascular Risk Factors

SBP tends to be a stronger predictor of BP-related outcomes than diastolic BP.[7-9] Long-term cumulative mean vascular risk factor levels have improved prediction of clinical outcomes compared with single measurements[10] means over discrete intervals (e.g., ≤1 year, 1 to 5 years) before outcome measurement,[8] and are associated with cognitive trajectories.[11] Glucose was treated as a continuous variable because higher blood glucose is a risk factor for dementia even in adults without diabetes.[12] We summarized post-stroke glucose as the time-dependent cumulative mean (or "running average") of all post-stroke glucose measurements before each cognitive assessment. This cumulative mean (is a running average) is calculated by adding up all of mean post-stroke glucose measures available to a given point it time and dividing by the number of available measures calculated by adding up all post-stroke glucose measures available at a given point in time and dividing by the number of available measures. We allowed measures of post-stroke vascular risk factors and cognition to occur on the same day because measured levels of vascular risk factors can plausibly be assumed to apply to the time preceding the measurement.

© 2023 Levine DA et al. *JAMA Network Open.*

The 4 cohorts instructed participants to fast before exams, except for the CHS year 7 exam. We defined fasting as abstaining from food for 8 or more hours. Infrequently, a participant did not fast 8 hours for a particular exam. The primary analysis used the observed glucose levels regardless of fasting status since the non-fasting glucose measurements still have information. A sensitivity analysis used estimated fasting glucose levels for non-fasting glucose measurements based on prior work.13

© 2023 Levine DA et al. *JAMA Network Open.*

Covariates

We harmonized covariates across cohorts by choosing common response categories for categorical variables and converting measurements to common units for continuous variables. Cohorts provided either fasting glucose levels or glucose levels with fasting indicators. We defined fasting as having gone 8 or more hours since eating. Glucose levels were treated as missing if not fasting. The cohorts used experts to adjudicate incident clinical events during follow-up (e.g., stroke, myocardial infarction, atrial fibrillation). History of atrial fibrillation was defined as a participant's reporting a history of atrial fibrillation at cohort baseline, cohort-adjudicated atrial fibrillation at baseline, or cohort-adjudicated incident atrial fibrillation between cohort baseline and stroke. History of myocardial infarction was defined as a participant's reporting a history of myocardial infarction at cohort baseline, cohort-adjudicated myocardial infarction at baseline, or cohort-adjudicated incident myocardial infarction between cohort baseline and stroke. Cohorts measured current hypertension, diabetes, and lipid-lowering medication use by evidence of medication bottles and/or self-report. Physical activity was defined as any physical activity in the past 2 weeks because one cohort had this information only. Kidney function (glomerular filtration rate) was measured using the Chronic Kidney Disease Epidemiology Collaboration (CKD-EPI) equation.

Depression risk is higher after stroke,[13] and post-stroke depressive symptoms are associated with cognitive dysfunction.[14] Depressive symptoms were measured by Center for Epidemiologic Studies Depression Scale (CES-D). Each cohort measured a version of the CES-D (ARIC used 12-item CES-D; CHS used 10-item CES-D for the first 8 exams and then a 2-item CES-D; FOS used a 20-item CES-D; REGARDS used a 10-item version of CES-D during follow-up). The CES-D scales were harmonized across the cohorts. For each cohort, we calculated an average CES-D score by summing the scores of the individual CES-D items divided by the number of CES-D items in the CES-D scale. Each cohort measured the apolipoprotein E (APOE) gene in a

subset of participants by study design. The number of APOE4 alleles was measured as follows: 0 alleles for 22 (E2/E2), 23 (E2/E3), 33 (E3/E3); 1 allele for 24 (E2/E4) or 34 (E3/E4); and 2 alleles for 44 (E4/E4).

Statistical Analysis Details

We followed a pre-specified analysis plan. We evaluated model assumptions by inspecting residual plots and accompanying normality statistics.

Dementia Measurement for Eligibility/Exclusion Criteria

Participants with dementia before stroke were not eligible for the study and excluded. Three cohorts (ARIC, CHS, FOS) measured incident dementia by physician-adjudication using standard diagnostic criteria, study-specific protocols, and all available data including in-person neuropsychological and neurologic assessments, telephone interviews (participant or informant), brain imaging, and medical record review. Participants from ARIC, CHS, and FOS were excluded if they had cohort-defined incident dementia before the first incident stroke during follow-up. One cohort (REGARDS) did not have incident dementia by physician-adjudication. REGARDS participants were excluded if they incident dementia before the first incident stroke during follow-up measured by a Six-Item Screener score <4 (three or more errors), which has a sensitivity and specificity for a diagnosis of dementia of 88.7% and 88.0%, respectively in a community-based sample of older Black adults.[15]

eFigure. Derivation of the Participant Cohort

Legend: Abbreviations. SBP=systolic blood pressure. We excluded participants reporting race other than White or Black because Reasons for Geographic And Racial Differences in Stroke Study (REGARDS)recruited White and Black participants by study design.

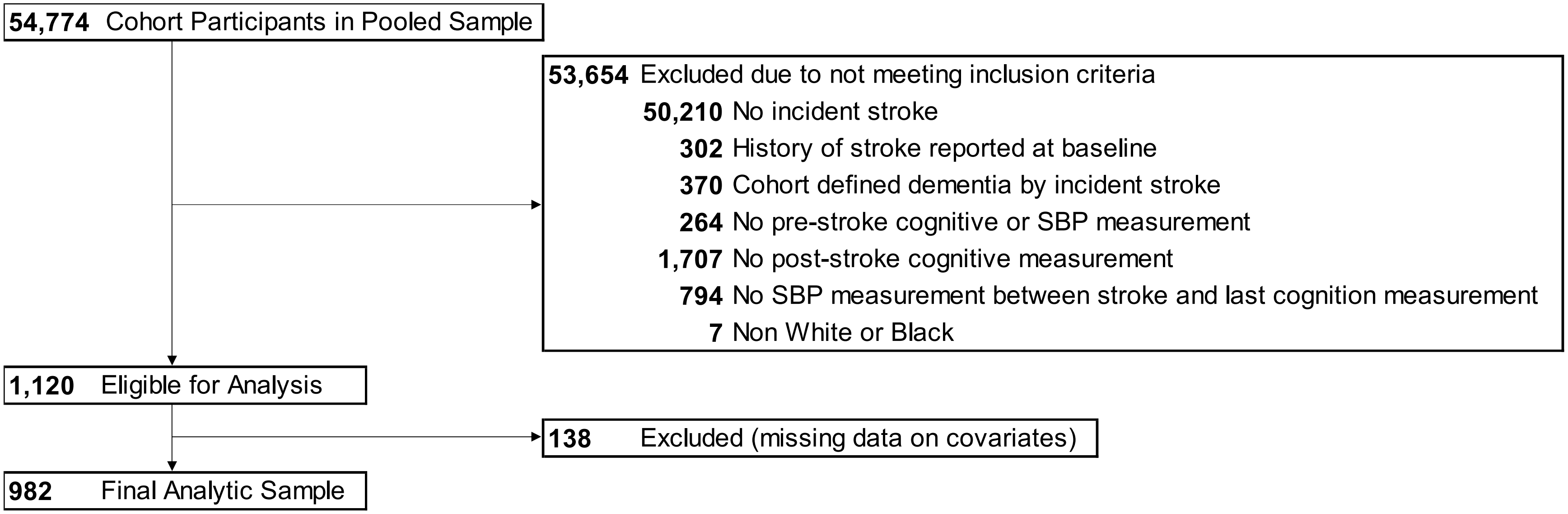

© 2023 Levine DA et al. *JAMA Network Open.*

eTable 1.[ab] Characteristics of Participants at First Poststroke Cognitive Assessment in the Pooled Cohort Sample by Cohort: STROKE COG Study, 1971 to 2021

Variable	ARIC No. (%)	CHS No. (%)	FOS No. (%)	REGARDS No. (%)
**Age at time of stroke, y**				
Range	48.7 to 91.9	66.2 to 94.9	44.1 to 89.5	50.2 to 96.4
Median (Q1, Q3)	71.7 (65.1, 78.3)	78.2 (74.0, 82.1)	71.2 (62.9, 77.0)	73.0 (67.5, 79.1)
**Measures at Cohort Baseline**				
Women	106 (44.5)	183 (55.1)	55 (54.5)	136 (43.7)
**Race/Ethnicity**				
Black	53 (22.3)	33 (9.9)	0 (0.0)	203 (65.3)
White	185 (77.7)	299 (90.1)	101 (100.0)	108 (34.7)
**Measures by the first post-stroke cognitive assessment**				
**Education**				
Less than high school	43 (18.1)	95 (28.6)	10 (9.9)	28 (9.0)
Completed high school	82 (34.4)	96 (28.9)	27 (26.7)	80 (25.7)
Some college but no degree	25 (10.5)	79 (23.8)	36 (35.7)	90 (29.0)
College graduate or more	88 (37.0)	62 (18.7)	28 (27.7)	113 (36.3)
**Income**				
Less than $5,000	6 (2.5)	19 (5.7)	0 (0.0)	3 (1.0)
$5,000 through $24,999	67 (28.1)	151 (45.5)	10 (9.9)	68 (21.9)
$25,000 through $34,999	34 (14.3)	43 (13.0)	23 (22.8)	63 (20.3)
$35,000 through $49,999	40 (16.8)	22 (6.6)	21 (20.8)	56 (18.0)
$50,000 and greater	58 (24.4)	41 (12.4)	22 (21.8)	85 (27.3)
Refused to answer/missing	33 (13.9)	56 (16.9)	25 (24.8)	36 (11.6)
Current cigarette smoking	27 (11.3)	21 (6.3)	12 (11.9)	45 (14.7)
Physical activity	137 (57.6)	282 (84.9)	77 (76.2)	231 (74.3)
Body mass index, kg/m	27.6 (24.7, 31.0)	26.2 (23.5, 29.3)	27.2 (24.7, 29.8)	27.9 (25.6, 31.8)
Waist circumference, cm	100.2 (92.3, 108.6)	96.0 (88.4, 104.3)	98.6 (90.2, 106.3)	96.5 (88.9, 105.4)
Alcoholic drinks per week	0 (0, 2)	0 (0, 0)	2 (0, 9)	0 (0, 1)
History of acute myocardial infarction	14 (5.9)	49 (14.8)	0 (0.0)	52 (16.7)
History of atrial fibrillation	0 (0.0)	18 (5.4)	0 (0.0)	38 (12.2)
Glomerular filtrate rate, mL/min	78.4 (63.1, 97.7)	60.9 (50.1, 71.7)	65.8 (53.9, 86.1)	82.0 (67.9, 93.2)

© 2023 Levine DA et al. *JAMA Network Open.*

Variable	ARIC No. (%)	CHS No. (%)	FOS No. (%)	REGARDS No. (%)
Pre-stroke SBP cumulative mean (SD), mmHg	146.7 (15.1)	148.4 (20.4)	131.0 (13.4)	130.2 (15.9)
Pre-stroke fasting glucose cumulative mean (SD), mg/dL	117.0 (42.5)	117.3 (40.9)	105.3 (22.5)	107.1 (44.3)
Pre-stroke LDL cholesterol cumulative mean (SD), mg/dL	130.7 (30.5)	132.1 (33.2)	138.2 (31.7)	113.1 (35.1)
Anti-hypertensive medication use, No. (%)	176 (74.0)	245 (73.8)	64 (63.4)	186 (59.8)
Diabetes medication use, No. (%)	68 (28.6)	55 (16.6)	15 (14.9)	65 (21.6)
Cholesterol medication use, No. (%)	149 (62.6)	47 (14.2)	57(56.4)	117 (38.2)
**Pre-stroke cognitive scores, median (Q1, Q3)**				
General cognitive performance	51.6 (46.5, 56.0)	49.1 (44.1, 54.2)	56.0 (53.1, 59.0)	55.0 (51.8, 57.6)
Executive function	51.8 (46.3, 56.3)	46.9 (41.4, 53.1)	54.1 (49.8, 54.5)	50.6 (44.9, 57.1)
Memory	52.9 (49.5, 56.4)	52.4 (49.1, 55.3)	53.9 (51.0, 57.4)	55.3 (53.2, 57.4)
**Number of pre-stroke cognitive assessments per individual, median (Q1, Q3)**				
Global cognitive performance	2 (1, 3)	4 (2, 7)	4 (2, 5)	6 (4, 9)
Executive function	2 (1, 3)	4 (2, 7)	4 (2, 5)	1 (0, 2)
Memory	2 (1, 2)	4 (2, 7)	4 (2, 5)	5 (3, 7)
**Post-stroke Measures**				
Follow-up time after stroke for primary outcome (global cognition), y	4.3 (2.1,10.2)	3.8 (1.9, 6.3)	6.0 (2.2, 10.8)	5.8 (3.9, 8.1)
SBP mean (SD) at first post-stroke cognitive assessment, mmHg	140.2 (23.3)	139.0 (22.9)	132.6 (21.9)	127.2 (16.8)
Number of SBP measurements after stroke	1 (1, 2)	2 (1, 4)	1 (1, 2)	1 (1, 1)
Time from stroke to first SBP measurement, y	2.7 (1.2, 5.5)	0.7 (0.4, 1.3)	1.8 (0.9, 3.3)	3.4 (1.4, 5.6)
Glucose mean (SD) at first post-stroke cognitive assessment, mg/dL	101.7 (20.2)	111.4 (37.1)	115.0 (31.0)	105.6 (35.9)
Number of glucose measurements after stroke	1 (0, 2)	1 (1, 2)	1 (1, 2)	1 (1, 1)
Time from stroke to first glucose measurement, y	2.2 (0.9, 4.6)	1.5 (0.7, 2.2)	1.8 (1.0, 3.4)	3.4 (1.5, 6.0)
LDL cholesterol mean (SD) at first post-stroke cognitive assessment, mg/dL	97.6 (33.6)	104.8 (43.7)	98.2 (34.1)	87.8 (33.0)
Number of LDL cholesterol measurements after stroke	1 (1, 2)	0 (0, 1)	1(1, 2)	1 (1, 1)

© 2023 Levine DA et al. *JAMA Network Open.*

Variable	ARIC	CHS	FOS	REGARDS
Time from stroke to first LDL cholesterol measurement, y	2.9 (1.2, 5.6)	2.1 (1.1, 3.6)	1.8 (1.0, 3.4)	3.4 (1.5, 6.0)
**Cognitive scores at first post-stroke cognitive assessment, median (Q1, Q3)**				
General cognitive performance	42.7 (35.3, 49.9)	45.5 (37.8, 51.1)	53.2 (47.6, 61.4)	58.5 (48.1, 58.5)
Executive function	43.2 (35.7, 49.8)	43.1 (36.1, 49.8)	54.1 (43.2, 54.1)	45.4 (40.8, 52.1)
Memory	44.1 (33.4, 51.7)	49.1 (43.5, 57.4)	57.4 (49.1, 57.4)	57.4 (49.1, 57.4)
**Number of post-stroke cognitive assessments per individual, median (Q1, Q3)**				
Global cognitive performance	1 (1, 2)	3 (2, 5)	2 (1, 3)	4 (3, 5)
Executive function	1 (1, 2)	3 (2, 5)	2 (1, 3)	1 (1, 2)
Memory	1 (1, 2)	2 (1, 4)	2 (1, 3)	3 (2, 3)

Abbreviations: ARIC=Atherosclerosis Risk in Communities Study. BP=blood pressure. CHS= Cardiovascular Health Study. LDL=low-density lipoprotein. FOS=Framingham Offspring Study. REGARDS=Reasons for Geographic And Racial Differences in Stroke Study. SD=standard deviation.
a: We excluded participants reporting race other than White or Black because REGARDS recruited White and Black participants by study design.
b: All cognitive measures are set to a T-score metric (mean 50, SD 10); a 1-point difference represents a 0.1 SD difference in the distribution of cognition across the 4 cohorts. Higher cognitive scores indicate better performance. P-value calculated from chi-square test for categorical variables and t-test for continuous variables.

© 2023 Levine DA et al. *JAMA Network Open.*

eTable 2.[ab] Descriptive Comparison of Included (n=982) vs Excluded (n=138) Participants

Variable	Included Participants No. (%)	Excluded Participants No. (%)	P[g]
**Age at time of stroke, y**			
Range	44.1 to 96.4	50.3 to 91.6	<0.001
Median (Q1, Q3)	74.6 (69.1, 79.8)	71.8 (66.0, 77.0)	
**Measures at Cohort Baseline**			
Women	480 (48.9)	75 (54.4)	0.23
**Race/Ethnicity**			
Black	289 (29.4)	48 (34.8)	0.20
White	693 (70.6)	90 (65.2)	
**Cohort**			
ARIC	238 (24.2)	17 (12.3)	<0.001
CHS	332 (33.8)	21 (15.2)	
FOS	101 (10.3)	13 (9.4)	
REGARDS	311 (31.7)	87 (63.0)	
**Measures by the First Post-Stroke Cognitive Assessment**			
**Education**			
Less than high school	176 (17.9)	26 (18.8)	<0.001
Completed high school	285 (29.0)	35 (25.4)	
Some college but no degree	230 (23.4)	32 (23.2)	
College graduate or more	291 (29.7)	39 (28.3)	
Refused to answer/missing	0 (0.0)	6 (4.3)	
**Income**			
Less than $5,000	28 (2.9)	0 (0.0)	0.03
$5,000 through $24,999	296 (30.1)	55 (39.9)	
$25,000 through $34,999	163 (16.6)	16 (11.6)	
$35,000 through $49,999	139 (14.1)	13 (9.4)	
$50,000 and greater	206 (21.0)	28 (20.3)	
Refused to answer/missing	150 (15.3)	26 (18.8)	
Current cigarette smoking	105 (10.7)	15 (10.9)	0.03
Physical activity	727 (74.0)	87 (63.0)	<0.001
Body mass index, kg/m	27.2 (24.6, 30.5)	28.2 (25.3, 31.8)	0.04
Waist circumference, cm	97.5 (89.6, 106.0)	97.0 (88.9, 106.7)	0.88
Alcoholic drinks per week	0 (0, 1)	0 (0, 1)	0.006
History of acute myocardial infarction	115 (11.7)	18 (13.0)	<0.001
History of atrial fibrillation	56 (5.7)	12 (8.7)	0.17
Glomerular filtrate rate, mL/min	71.7 (56.8, 88.3)	84.8 (65.3, 98.5)	<0.001
Pre-stroke SBP, mean (SD), mm Hg	140.4 (19.2)	138.8 (24.6)	0.47
Pre-stroke fasting glucose, mean (SD), mg/dL	112.8 (41.2)	118.7 (47.4)	0.18
Pre-stroke LDL cholesterol, mean (SD), mg/dL	126.4 (34.2)	128.8 (39.4)	0.52
Anti-hypertensive medication use	671 (68.3)	87 (63.0)	<0.001
Diabetes medication use	203 (20.7)	43 (31.2)	0.003
Cholesterol medication use	370 (37.7)	49 (35.5)	0.85

© 2023 Levine DA et al. *JAMA Network Open.*

Variable	Included Participants	Excluded Participants	*P*
**Pre-stroke cognitive scores, median (Q1, Q3)**			
General cognitive performance	52.7 (47.9, 56.7)	52.4 (49.1, 56.1)	0.80
Executive function	50.3 (44.0, 55.2)	49.4 (43.2, 55.2)	0.33
Memory	54.1 (50.1, 56.6)	53.2 (49.6, 57.0)	0.36
**Number of pre-stroke cognitive assessments per individual, median (Q1, Q3)**			
General cognitive performance	4 (2,7)	4 (2,7)	0.23
Executive function	2 (1,4)	2 (1,3)	<0.001
Memory	3 (2,6)	3 (2,5)	0.42
**Post-Stroke Measures**			
Follow-up time after stroke for primary outcome (global cognition), y	4.7 (2.6, 7.9)	6.9 (3.8, 10.1)	0.003
SBP mean (SD) at first post-stroke cognitive assessment, mmHg	134.9 (21.9)	143.2 (27.4)	0.05
Number of SBP measurements after stroke	1 (1, 2)	1 (1, 1)	<0.001
Time from stroke to first SBP measurement, y	1.6 (0.7, 4.1)	3.6 (1.5, 6.2)	<0.001
Glucose mean (SD) at first post-stroke cognitive assessment, mg/dL	108.1 (34.4)	117.7 (45.1)	0.40
Number of glucose measurements after stroke	1 (1, 1)	1 (0, 1)	<0.001
Time from stroke to first glucose measurement, y	2.0 (0.9, 4.0)	3.6 (1.8, 5.7)	<0.001
LDL cholesterol mean (SD) at first post-stroke cognitive assessment, mg/dL	94.1 (34.9)	98.0 (34.9)	0.59
Number of LDL cholesterol measurements after stroke	1 (0, 1)	1 (0, 1)	0.12
Time from stroke to first LDL cholesterol measurement, y	2.6 (1.2, 5.0)	3.9 (2.2, 6.7)	0.004
**Cognitive scores at first post-stroke cognitive assessment, median (Q1, Q3)**			
General cognitive performance	49.1 (40.2, 58.2)	55.7 (48.1, 58.5)	<0.001
Executive function	44.1 (37.5, 52.5)	45.4 (41.2, 52.1)	0.37
Memory	49.1 (43.5, 57.4)	57.4 (49.1, 57.4)	<0.001
**Number of post-stroke cognitive assessments per individual, median (Q1, Q3)**			
Global cognitive performance	3 (1, 4)	2 (0, 7)	0.03
Executive function	2 (1, 3)	1 (0, 2)	<0.001
Memory	2 (1, 3)	2 (0, 5)	0.24

Abbreviations: ARIC=Atherosclerosis Risk in Communities Study. CHS= Cardiovascular Health Study. LDL=low-density lipoprotein. FOS=Framingham Offspring Study. REGARDS=Reasons for Geographic And Racial Differences in Stroke Study. SBP=systolic blood pressure. SD=standard deviation.
a: We excluded participants reporting race other than White or Black because REGARDS recruited White and Black participants by study design.
b: All cognitive measures are set to a T-score metric (mean 50, SD 10); a 1-point difference represents a 0.1 SD difference in the distribution of cognition across the 4 cohorts. Higher cognitive scores indicate better performance. P-value calculated from chi-square test for categorical variables and t-test for continuous variables.

© 2023 Levine DA et al. *JAMA Network Open.*

eTable 3.[abc] Sensitivity Analysis of Association of Poststroke Vascular Risk Factor Levels With Poststroke Executive Function Decline Including Number of APOE4 Alleles Among Participants With APOE4 Information: STROKE COG Study, 1971 to 2021

	Model M1a: Time-varying post-stroke systolic BP (n=711)		Model M1b: Time-varying post-stroke glucose (n=546)		Model M1c: Time-varying post-stroke LDL cholesterol (n=499)		Model M2: Joint time-varying post-stroke systolic BP, glucose, and LDL cholesterol (n=397)	
**Coefficient**	**Estimate (95% CI)**	***P***	**Estimate (95% CI)**	***P***	**Estimate (95% CI)**	***P***	**Estimate (95% CI)**	***P***
Slope (change in cognition over time), per year	-0.42 (-0.64 to -0.20)	<0.001	-0.13 (-0.35 to 0.08)	0.23	-0.29 (-0.50 to -0.08)	<0.01	-0.40 (-0.67 to -0.12)	0.005
Effect of age at stroke (per 10-year increase) on slope, per year	-0.12 (-0.24 to 0.01)	0.03	-0.05 (-0.17 to 0.08)	0.49	-0.07 (-0.19 to 0.04)	0.22	-0.09 (-0.22 to 0.04)	0.16
Effect of female sex at stroke on slope, per year	-0.18 (-0.38 to 0.02)	0.08	-0.29 (-0.52 to -0.05)	0.02	-0.18 (-0.40 to 0.04)	0.11	-0.30 (-0.54 to -0.05)	0.02
Effect of 1 APOE4 allele vs 0 APOE4 alleles on slope, per year	-0.15 (-0.39 to 0.08)	0.20	-0.31 (-0.58 to -0.03)	0.03	-0.17 (-0.42 to 0.08)	0.19	-0.21 (-0.49 to 0.06)	0.13
Effect of 2 APOE4 alleles vs 0 APOE4 alleles on slope, per year	-1.01 (-1.78 to -0.24)	0.01	-1.20 (-2.08 to -0.32)	0.008	-1.15 (-1.96 to -0.34)	0.005	-1.43 (-2.32 to -0.54)	0.002
Effect of post-stroke systolic BP (per 10 mm Hg increase) on slope, per year	0.002 (-0.05 to 0.06)	0.95	NA	NA	NA	NA	-0.01 (-0.08 to 0.07)	0.89
Effect of post-stroke glucose (per 10 mg/dL increase) on slope, per year	NA	NA	-0.02 (-0.06 to 0.03)	0.46	NA	NA	-0.02 (-0.06 to 0.03)	0.5
Effect of post-stroke LDL cholesterol (per 10 mg/dL increase) on slope, per year	NA	NA	NA	NA	0.002 (-0.03 to 0.04)	0.91	-0.003 (-0.04 to 0.04)	0.87

Abbreviations: BP=blood pressure. LDL=low-density lipoprotein. NA=not applicable.
a: All cognitive measures are set to a T-score metric (mean 50, SD 10); a 1-point difference represents a 0.1 SD difference in the distribution of cognition across the 4 cohorts. Higher cognitive scores indicate better performance.

© 2023 Levine DA et al. *JAMA Network Open.*

b: Linear mixed-effects models included number of APOE4 alleles, time since stroke, race, sex, age at time of stroke, cohort, education, income, medication for hypertension, diabetes, and high cholesterol, pre-stroke body mass index, waist circumference, smoking status, physical activity, alcohol consumption per week, history of MI, history of atrial fibrillation, glomerular filtration rate, cohort study, pre-stroke mean executive function, pre-stroke mean systolic BP, pre-stroke mean glucose, pre-stroke mean LDL, post-stroke mean systolic BP, post-stroke mean glucose, post-stroke mean LDL, age at time of stroke*time since stroke, sex*time since stroke, post-stroke mean systolic BP*time since stroke, post-stroke mean glucose*time since stroke, post-stroke mean LDL*time since stroke, anti-hypertensive medication use*time, anti-hyperglycemic medication use*time, and lipid lowering medication use*time. To consider correlation between longitudinal global cognition measures, we included random intercept and slope effect associated with participants. Glucose, LDL cholesterol, and systolic BP values are divided by 10 so that the parameter estimates refer to a 10-unit change in the variables. Each cognitive outcome is set to missing (censored) at the time of second expert-adjudicated incident stroke, death, loss to follow-up, or the end of follow-up, whichever occurs first. Models M1a, M1b, and M1c estimate the individual effect of post-stroke time-varying mean systolic BP, glucose, and LDL cholesterol on post-stroke executive function decline with separate models. Model M1a includes a post-stroke time-varying mean systolic BP level by time interaction and post-stroke time-varying mean systolic BP. Model M1b includes a post-stroke time-varying mean glucose level by time interaction and post-stroke time-varying mean glucose. Model M1c includes post-stroke time-varying mean LDL cholesterol level by time interaction and post-stroke time-varying mean LDL cholesterol. Model M2 estimates the joint effect of post-stroke time-varying mean systolic BP, glucose, and LDL cholesterol on post-stroke executive function decline. Model M2 includes the post-stroke time-varying mean systolic BP, glucose, and LDL cholesterol and their interactions with time.
c: Median (interquartile range) number of executive function assessments before stroke was 2 (2, 5) and after stroke was 2 (1, 3).

© 2023 Levine DA et al. *JAMA Network Open.*

eTable 4. Sensitivity Analysis of Association of Poststroke Vascular Risk Factor Levels With Poststroke Memory[ac] Decline Including Number of APOE4 Alleles Among Participants With APOE4 Information: STROKE COG Study, 1971 to 2019

	Model[b] M1a: Time-varying post-stroke systolic BP (n=757)		Model M1b: Time-varying post-stroke glucose (n=587)		Model M1c: Time-varying post-stroke LDL cholesterol (n=532)		Model M2: Joint time-varying post-stroke systolic BP, glucose, and LDL cholesterol (n=420)	
**Coefficient**	**Estimate (95% CI)**	***P***	**Estimate (95% CI)**	***P***	**Estimate (95% CI)**	***P***	**Estimate (95% CI)**	***P***
Slope (change in cognition over time), per year	-0.16 (-0.40 to 0.08)	0.20	-0.24 (-0.46 to -0.02)	0.04	-0.22 (-0.46 to 0.03)	0.08	-0.36 (-0.69 to -0.04)	0.03
Effect of age at stroke (per 10-year increase) on slope, per year	-0.03 (-0.16 to 0.10)	0.62	-0.02 (-0.16 to 0.12)	0.79	-0.08 (-0.23 to 0.07)	0.28	-0.03 (-0.20 to 0.14)	0.72
Effect of female sex at stroke on slope, per year	-0.28 (-0.51 to -0.06)	0.01	-0.30 (-0.55 to -0.05)	0.02	-0.33 (-0.59 to -0.06)	0.01	-0.31 (-0.60 to -0.01)	0.04
Effect of 1 APOE4 allele vs 0 APOE4 alleles on slope, per year	-0.30 (-0.55 to -0.04)	0.02	-0.17 (-0.45 to 0.11)	0.23	-0.30 (-0.60 to -0.01)	0.046	-0.13 (-0.47 to 0.21)	0.46
Effect of 2 APOE4 alleles vs 0 APOE4 alleles on slope, per year	-0.29 (-1.00 to 0.42)	0.43	-0.28 (-1.01 to 0.45)	0.45	-0.27 (-1.05 to 0.51)	0.50	-0.46 (-1.29 to 0.37)	0.28
Effect of post-stroke systolic BP (per 10 mm Hg increase) on slope, per year	0.02 (-0.04 to 0.08)	0.51	NA	NA	NA	NA	0.002 (-0.09 to 0.09)	0.97
Effect of post-stroke glucose (per 10 mg/dL increase) on slope, per year	NA	NA	-0.002 (-0.05 to 0.04)	0.93	NA	NA	-0.0001 (-0.06 to 0.06)	0.99
Effect of post-stroke LDL cholesterol (per 10 mg/dL increase) on slope, per year	NA	NA	NA	NA	-0.0002 (-0.04 to 0.04)	0.99	0.01 (-0.04 to 0.05)	0.82

© 2023 Levine DA et al. *JAMA Network Open.*

Abbreviations: BP=blood pressure. LDL=low-density lipoprotein. NA=not applicable.
a: All cognitive measures are set to a T-score metric (mean 50, SD 10); a 1-point difference represents a 0.1 SD difference in the distribution of cognition across the 4 cohorts. Higher cognitive scores indicate better performance.
b: Linear mixed-effects models included number of APOE4 alleles, time since stroke, race, sex, age at time of stroke, cohort, education, income, medication for hypertension, diabetes, and high cholesterol, pre-stroke BMI, waist circumference, smoking status, physical activity, alcohol consumption per week, history of MI, history of atrial fibrillation, glomerular filtration rate, cohort study, pre-stroke mean memory, pre-stroke mean systolic BP, pre-stroke mean glucose, pre-stroke mean LDL, post-stroke mean systolic BP, post-stroke mean glucose, post-stroke mean LDL, age at time of stroke*time since stroke, sex*time since stroke, post-stroke mean systolic BP*time since stroke, post-stroke mean glucose*time since stroke, post-stroke mean LDL*time since stroke, anti-hypertensive medication use*time, anti-hyperglycemic medication use*time, and lipid lowering medication use*time. To consider correlation between longitudinal global cognition measures, we included random intercept and slope effect associated with participants. Glucose, LDL cholesterol, and systolic BP values are divided by 10 so that the parameter estimates refer to a 10-unit change in the variables. Each cognitive outcome is set to missing (censored) at the time of second expert-adjudicated incident stroke, death, loss to follow-up, or the end of follow-up, whichever occurs first. Models M1a, M1b, and M1c estimate the individual effect of post-stroke time-varying mean systolic BP, glucose, and LDL cholesterol on post-stroke memory decline with separate models. Model M1a includes a post-stroke time-varying mean systolic BP level by time interaction and post-stroke time-varying mean systolic BP. Model M1b includes a post-stroke time-varying mean glucose level by time interaction and post-stroke time-varying mean glucose. Model M1c includes post-stroke time-varying mean LDL cholesterol level by time interaction and post-stroke time-varying mean LDL cholesterol. Model M2 estimates the joint effect of post-stroke time-varying mean systolic BP, glucose, and LDL cholesterol on post-stroke memory decline. Model M2 includes the post-stroke time-varying mean systolic BP, glucose, and LDL cholesterol and their interactions with time.
c: Median (interquartile range) number of memory assessments before stroke was 3 (2, 5) and after stroke was 2 (1, 3).

© 2023 Levine DA et al. *JAMA Network Open.*

eTable 5. Sensitivity Analysis of Association of Poststroke Time-Invariant Mean Vascular Risk Factor Levels and Poststroke Global Cognition[ac] Decline: STROKE COG Study, 1971 to 2021

	Model[b] M1a: Time-invariant post-stroke systolic BP (n=1022)		Model M1b: Time-invariant post-stroke glucose (n=830)		Model M1c: Time-invariant post-stroke LDL cholesterol (n=770)		Model M2: Joint time-invariant post-stroke systolic BP, glucose, and LDL cholesterol (n=646)	
**Coefficient**	**Estimate (95% CI)**	***P***	**Estimate (95% CI)**	***P***	**Estimate (95% CI)**	***P***	**Estimate (95% CI)**	***P***
Slope (change in cognition over time), per year	-0.33 (-0.48 to -0.18)	<0.001	-0.25 (-0.38 to -0.12)	<0.001	-0.26 (-0.41 to -0.12)	<0.001	-0.34 (-0.51 to -0.18)	<0.001
Effect of age at stroke (per 10-year increase) on slope, per year	-0.21 (-0.31 to -0.12)	<0.001	-0.20 (-0.30 to -0.11)	<0.001	-0.20 (-0.30 to -0.10)	<0.001	-0.20 (-0.30 to -0.10)	<0.001
Effect of female sex at stroke on slope, per year	-0.15 (-0.31 to 0.01)	0.06	-0.20 (-0.36 to -0.03)	0.02	-0.15 (-0.32 to 0.02)	0.09	-0.23 (-0.40 to -0.05)	0.01
Effect of post-stroke systolic BP (per 10 mm Hg increase) on slope, per year	-0.004 (-0.05 to 0.04)	0.85	NA	NA	NA	NA	0.004 (-0.05 to 0.06)	0.89
Effect of post-stroke glucose (per 10 mg/dL increase) on slope, per year	NA	NA	-0.05 (-0.07 to -0.02)	<0.001	NA	NA	-0.06 (-0.09 to -0.03)	<0.001
Effect of post-stroke LDL cholesterol (per 10 mg/dL increase) on slope, per year	NA	NA	NA	NA	-0.01 (-0.04 to 0.01)	0.38	-0.01 (-0.04 to 0.02)	0.50

© 2023 Levine DA et al. *JAMA Network Open.*

Abbreviations: BP=blood pressure. LDL=low-density lipoprotein. NA=not applicable.
a: Global cognitive performance measures global cognition. All cognitive measures are set to a T-score metric (mean 50, SD 10); a 1-point difference represents a 0.1 SD difference in the distribution of cognition across the 4 cohorts. Higher cognitive scores indicate better performance.
b: Linear mixed-effects models included time since stroke, race, sex, age at time of stroke, cohort, education, income, medication for hypertension, diabetes, and high cholesterol, pre-stroke body mass index, waist circumference, smoking status, physical activity, alcohol consumption per week, history of MI, history of atrial fibrillation, glomerular filtration rate, cohort study, pre-stroke mean global cognition, pre-stroke mean systolic BP, pre-stroke mean glucose, pre-stroke mean LDL, post-stroke mean systolic BP, post-stroke mean glucose, post-stroke mean LDL, age at time of stroke*time since stroke, sex*time since stroke, post-stroke mean systolic BP*time since stroke, post-stroke mean glucose*time since stroke, post-stroke mean LDL*time since stroke, anti-hypertensive medication use*time, anti-hyperglycemic medication use*time, and lipid lowering medication use*time. To consider correlation between longitudinal global cognition measures, we included random intercept and slope effect associated with participants. Glucose, LDL cholesterol, and systolic BP values are divided by 10 so that the parameter estimates refer to a 10-unit change in the variables. Each cognitive outcome is set to missing (censored) at the time of second expert-adjudicated incident stroke, death, loss to follow-up, or the end of follow-up, whichever occurs first. Models M1a, M1b, and M1c estimate the individual effect of post-stroke time-invariant mean systolic BP, glucose, and LDL cholesterol levels on global cognitive decline with separate models. Model M1a includes a post-stroke time-invariant mean systolic BP level by time interaction and post-stroke time-invariant mean systolic BP. Model M1b includes a post-stroke time-invariant mean glucose level by time interaction and post-stroke time-invariant mean glucose. Model M1c includes post-stroke time-invariant mean LDL cholesterol level by time interaction and post-stroke time-invariant mean LDL cholesterol. Model M2 estimates the joint effect of post-stroke time-invariant mean systolic BP, glucose, and LDL cholesterol) on post-stroke global cognitive decline. Model M2 includes the post-stroke time-invariant mean systolic BP, glucose, and LDL cholesterol and their interactions with time.
c: Median (interquartile range) number of global cognition assessments before stroke was 4 (2, 7) and after stroke was 3 (2, 7).

© 2023 Levine DA et al. *JAMA Network Open.*

eTable 6. Association of Poststroke Invariant Mean Vascular Risk Factor Levels and Poststroke Executive Function[ac] Decline: STROKE COG Study, 1971 to 2021

	Model[b] M1a: Time-invariant post-stroke systolic BP (n=899)		Model M1b: Time-invariant post-stroke glucose (n=718)		Model M1c: Time-invariant post-stroke LDL cholesterol (n=658)		Model M2: Joint time-invariant post-stroke systolic BP, glucose, and LDL cholesterol (n=541)	
**Coefficient**	**Estimate (95% CI)**	***P***	**Estimate (95% CI)**	***P***	**Estimate (95% CI)**	***P***	**Estimate (95% CI)**	***P***
Slope (change in cognition over time), per year	-0.52 (-0.72 to -0.32)	<0.001	-0.43 (-0.61 to -0.26)	<0.001	-0.42 (-0.60 to -0.23)	<0.001	-0.60 (-0.82 to -0.38)	<0.001
Effect of age at stroke (per 10-year increase) on slope, per year	-0.01 (-0.22 to -0.00)	0.04	-0.07 (-0.19 to 0.04)	0.22	-0.09 (-0.20 to 0.02)	0.10	-0.08 (-0.20 to 0.04)	0.17
Effect of female sex at stroke on slope, per year	-0.16 (-0.35 to 0.04)	0.11	-0.09 (-0.31 to 0.12)	0.40	-0.11 (-0.31 to 0.09)	0.29	-0.12 (-0.33 to 0.09)	0.27
Effect of post-stroke systolic BP (per 10 mm Hg increase) on slope, per year	-0.02 (-0.07 to 0.04)	0.52	NA	NA	NA	NA	-0.01 (-0.08 to 0.05)	0.69
Effect of post-stroke glucose (per 10 mg/dL increase) on slope, per year	NA	NA	0.003 (-0.04 to 0.04)	0.88	NA	NA	-0.001 (-0.04 to 0.04)	0.95
Effect of post-stroke LDL cholesterol (per 10 mg/dL increase) on slope, per year	NA	NA	NA	NA	0.0002 (-0.03 to 0.03)	0.99	-0.01 (-0.04 to 0.03)	0.77

© 2023 Levine DA et al. *JAMA Network Open.*

Abbreviations: BP=blood pressure. LDL=low-density lipoprotein. NA=not applicable.
a: All cognitive measures are set to a T-score metric (mean 50, SD 10); a 1-point difference represents a 0.1 SD difference in the distribution of cognition across the 4 cohorts. Higher cognitive scores indicate better performance.
b: Linear mixed-effects models included time since stroke, race, sex, age at time of stroke, cohort, education, income, medication for hypertension, diabetes, and high cholesterol, pre-stroke body mass index, waist circumference, smoking status, physical activity, alcohol consumption per week, history of MI, history of atrial fibrillation, glomerular filtration rate, cohort study, pre-stroke mean executive function, pre-stroke mean systolic BP, pre-stroke mean glucose, pre-stroke mean LDL, post-stroke mean systolic BP, post-stroke mean glucose, post-stroke mean LDL, age at time of stroke*time since stroke, sex*time since stroke, post-stroke mean systolic BP*time since stroke, post-stroke mean glucose*time since stroke, post-stroke mean LDL*time since stroke, anti-hypertensive medication use*time, anti-hyperglycemic medication use*time, and lipid lowering medication use*time. To consider correlation between longitudinal global cognition measures, we included random intercept and slope effect associated with participants. Glucose, LDL cholesterol, and systolic BP values are divided by 10 so that the parameter estimates refer to a 10-unit change in the variables. Each cognitive outcome is set to missing (censored) at the time of second expert-adjudicated incident stroke, death, loss to follow-up, or the end of follow-up, whichever occurs first. Models M1a, M1b, and M1c estimate the individual effect of post-stroke time-invariant mean systolic BP, glucose, and LDL cholesterol on post-stroke executive function decline with separate models. Model M1a includes a post-stroke time-invariant mean systolic BP level by time interaction and post-stroke time-invariant mean systolic BP. Model M1b includes a post-stroke time-invariant mean glucose level by time interaction and post-stroke time-invariant mean glucose. Model M1c includes post-stroke time-invariant mean LDL cholesterol level by time interaction and post-stroke time-invariant mean LDL cholesterol. Model M2 estimates the joint effect of post-stroke time-invariant mean systolic BP, glucose, and LDL cholesterol on post-stroke executive function decline. Model M2 includes the post-stroke time-invariant mean systolic BP, glucose, and LDL cholesterol and their interactions with time.
c: Median (interquartile range) number of executive function assessments before stroke was 2 (1, 4) and after stroke was 2 (1, 4).

© 2023 Levine DA et al. *JAMA Network Open.*

eTable 7. Association of Poststroke Time-Invariant Mean Vascular Risk Factor Levels and Poststroke Memory[ac] Decline: STROKE COG Study, 1971 to 2021

	Model[b] M1a: Time-invariant post-stroke systolic BP (n=975)		Model M1b: Time-invariant post-stroke glucose (n=791)		Model M1c: Time-invariant post-stroke LDL cholesterol (n=730)		Model M2: Joint time-invariant post-stroke systolic BP, glucose, and LDL cholesterol (n=608)	
**Coefficient**	**Estimate (95% CI)**	***P***	**Estimate (95% CI)**	***P***	**Estimate (95% CI)**	***P***	**Estimate (95% CI)**	***P***
Slope (change in cognition over time), per year	-0.11 (-0.28 to 0.05)	0.19	-0.14 (-0.28 to -0.01)	0.04	-0.07 (-0.23 to 0.08)	0.34	-0.09 (-0.28 to 0.09)	0.31
Effect of age at stroke (per 10-year increase) on slope, per year	-0.002 (-0.10 to 0.10)	0.97	-0.02 (-0.12 to 0.08)	0.67	-0.04 (-0.15 to 0.06)	0.44	-0.01 (-0.12 to 0.10)	0.83
Effect of female sex at stroke on slope, per year	-0.17 (-0.34 to 0.01)	0.06	-0.17 (-0.35 to 0.001)	0.05	-0.20 (-0.39 to -0.02)	0.03	-0.22 (-0.41 to -0.03)	0.03
Effect of post-stroke systolic BP (per 10 mm Hg increase) on slope, per year	-0.02 (-0.07 to 0.02)	0.34	NA	NA	NA	NA	-0.02 (-0.08 to 0.04)	0.43
Effect of post-stroke glucose (per 10 mg/dL increase) on slope, per year	NA	NA	-0.02 (-0.04 to 0.01)	0.28	NA	NA	-0.03 (-0.06 to 0.01)	0.12
Effect of post-stroke LDL cholesterol (per 10 mg/dL increase) on slope, per year	NA	NA	NA	NA	-0.01 (-0.04 to 0.02)	0.41	0.004 (-0.03 to 0.03)	0.79

© 2023 Levine DA et al. *JAMA Network Open.*

Abbreviations: BP=blood pressure. LDL=low-density lipoprotein. NA=not applicable.
a: All cognitive measures are set to a T-score metric (mean 50, SD 10); a 1-point difference represents a 0.1 SD difference in the distribution of cognition across the 4 cohorts. Higher cognitive scores indicate better performance.
b: Linear mixed-effects models included time since stroke, race, sex, age at time of stroke, cohort, education, income, medication for hypertension, diabetes, and high cholesterol, pre-stroke BMI, waist circumference, smoking status, physical activity, alcohol consumption per week, history of MI, history of atrial fibrillation, glomerular filtration rate, cohort study, pre-stroke mean memory, pre-stroke mean systolic BP, pre-stroke mean glucose, pre-stroke mean LDL, post-stroke mean systolic BP, post-stroke mean glucose, post-stroke mean LDL, age at time of stroke*time since stroke, sex*time since stroke, post-stroke mean systolic BP*time since stroke, post-stroke mean glucose*time since stroke, post-stroke mean LDL*time since stroke, anti-hypertensive medication use*time, anti-hyperglycemic medication use*time, and lipid lowering medication use*time. To consider correlation between longitudinal global cognition measures, we included random intercept and slope effect associated with participants. Glucose, LDL cholesterol, and systolic BP values are divided by 10 so that the parameter estimates refer to a 10-unit change in the variables. Each cognitive outcome is set to missing (censored) at the time of second expert-adjudicated incident stroke, death, loss to follow-up, or the end of follow-up, whichever occurs first. Models M1a, M1b, and M1c estimate the individual effect of post-stroke time-invariant mean systolic BP, glucose, and LDL cholesterol on post-stroke memory decline with separate models. Model M1a includes a post-stroke time-invariant mean systolic BP level by time interaction and post-stroke time-invariant mean systolic BP. Model M1b includes a post-stroke time-invariant mean glucose level by time interaction and post-stroke time-invariant mean glucose. Model M1c includes post-stroke time-invariant mean LDL cholesterol level by time interaction and post-stroke time-invariant mean LDL cholesterol. Model M2 estimates the joint effect of post-stroke time-invariant mean systolic BP, glucose, and LDL cholesterol on post-stroke memory decline. Model M2 includes the post-stroke time-invariant mean systolic BP, glucose, and LDL cholesterol and their interactions with time.
c: Median (interquartile range) number of memory assessments before stroke was 3 (2, 5) and after stroke was 3 (1, 5).

© 2023 Levine DA et al. *JAMA Network Open.*

eTable 8. Sensitivity Analysis of Association of Poststroke Vascular Risk Factor Levels and Poststroke Global Cognition[ac] Decline Including Participants With History of Stroke at Cohort Baseline: STROKE COG Study, 1971 to 2021

	Model[b] M1a: Time-varying post-stroke systolic BP (n=1017)		Model M1b: Time-varying post-stroke glucose (n=820)		Model M1c: Time-varying post-stroke LDL cholesterol (n=772)		Model M2: Joint time-invariant post-stroke systolic BP, glucose, and LDL cholesterol (n=637)	
**Coefficient**	**Estimate (95% CI)**	***P***	**Estimate (95% CI)**	***P***	**Estimate (95% CI)**	***P***	**Estimate (95% CI)**	***P***
Slope (change in cognition over time), per year	-0.49 (-0.68 to -0.30)	<0.001	-0.38 (-0.55 to -0.21)	<0.001	-0.43 (-0.61 to -0.25)	<0.001	-0.55 (-0.78 to -0.33)	<0.001
Effect of age at stroke (per 10-year increase) on slope, per year	-0.25 (-0.35 to -0.14)	<0.001	-0.18 (-0.30 to -0.07)	0.002	-0.21 (-0.32 to -0.09)	<0.001	-0.19 (-0.31 to -0.07)	0.002
Effect of female sex at stroke on slope, per year	-0.18 (-0.36 to 0.01)	0.06	-0.25 (-0.46 to -0.05)	0.02	-0.20 (-0.40 to 0.01)	0.06	-0.24 (-0.46 to -0.02)	0.03
Effect of post-stroke systolic BP (per 10 mm Hg increase) on slope, per year	0.03 (-0.01 to 0.08)	0.16	NA	NA	NA	NA	0.04 (-0.02 to 0.11)	0.20
Effect of post-stroke glucose (per 10 mg/dL increase) on slope, per year	NA	NA	-0.03 (-0.06 to 0.01)	0.15	NA	NA	-0.04 (-0.08 to -0.0003)	0.048
Effect of post-stroke LDL cholesterol (per 10 mg/dL increase) on slope, per year	NA	NA	NA	NA	0.01 (-0.02 to 0.04)	0.49	0.01 (-0.03 to 0.04)	0.75

© 2023 Levine DA et al. *JAMA Network Open.*

Abbreviations: BP=blood pressure. LDL=low-density lipoprotein. NA=not applicable.
a: Global cognitive performance measures global cognition. All cognitive measures are set to a T-score metric (mean 50, SD 10); a 1-point difference represents a 0.1 SD difference in the distribution of cognition across the 4 cohorts. Higher cognitive scores indicate better performance.
b: Linear mixed-effects models included time since stroke, race, sex, age at time of stroke, cohort, education, income, medication for hypertension, diabetes, and high cholesterol, pre-stroke body mass index, waist circumference, smoking status, physical activity, alcohol consumption per week, history of MI, history of atrial fibrillation, glomerular filtration rate, cohort study, pre-stroke mean global cognition, pre-stroke mean systolic BP, pre-stroke mean glucose, pre-stroke mean LDL, post-stroke mean systolic BP, post-stroke mean glucose, post-stroke mean LDL, age at time of stroke*time since stroke, sex*time since stroke, post-stroke mean systolic BP*time since stroke, post-stroke mean glucose*time since stroke, post-stroke mean LDL*time since stroke, anti-hypertensive medication use*time, anti-hyperglycemic medication use*time, and lipid lowering medication use*time. To consider correlation between longitudinal global cognition measures, we included random intercept and slope effect associated with participants. Glucose, LDL cholesterol, and systolic BP values are divided by 10 so that the parameter estimates refer to a 10-unit change in the variables. Each cognitive outcome is set to missing (censored) at the time of second expert-adjudicated incident stroke, death, loss to follow-up, or the end of follow-up, whichever occurs first. Models M1a, M1b, and M1c estimate the individual effect of post-stroke time-invariant mean systolic BP, glucose, and LDL cholesterol levels on global cognitive decline with separate models. Model M1a includes a post-stroke time-invariant mean systolic BP level by time interaction and post-stroke time-invariant mean systolic BP. Model M1b includes a post-stroke time-invariant mean glucose level by time interaction and post-stroke time-invariant mean glucose. Model M1c includes post-stroke time-invariant mean LDL cholesterol level by time interaction and post-stroke time-invariant mean LDL cholesterol. Model M2 estimates the joint effect of post-stroke time-invariant mean systolic BP, glucose, and LDL cholesterol) on post-stroke global cognitive decline. Model M2 includes the post-stroke time-invariant mean systolic BP, glucose, and LDL cholesterol and their interactions with time.
c: Median (interquartile range) number of global cognition assessments before stroke was 4 (2, 7) and after stroke was 3 (1, 4).

© 2023 Levine DA et al. *JAMA Network Open.*

eTable 9. Association of Poststroke Invariant Mean Vascular Risk Factor Levels and Poststroke Executive Function[ac] Decline Including Participants With History of Stroke at Cohort Baseline: STROKE COG Study, 1971 to 2021

	Model[b] M1a: Time-varying post-stroke systolic BP (n=875)		Model M1b: Time-varying post-stroke glucose (n=688)		Model M1c: Time-varying post-stroke LDL cholesterol (n=634)		Model M2: Joint time-varying post-stroke systolic BP, glucose, and LDL cholesterol (n=513)	
**Coefficient**	**Estimate (95% CI)**	***P***	**Estimate (95% CI)**	***P***	**Estimate (95% CI)**	***P***	**Estimate (95% CI)**	***P***
Slope (change in cognition over time), per year	-0.47 (-0.68 to -0.27)	<0.001	-0.32 (-0.50 to -0.13)	0.001	-0.35 (-0.55 to -0.16)	<0.001	-0.52 (-0.78 to -0.27)	<0.001
Effect of age at stroke (per 10-year increase) on slope, per year	-0.09 (-0.20 to 0.02)	0.11	-0.01 (-0.14 to 0.11)	0.83	-0.07 (-0.18 to 0.05)	0.25	-0.06 (-0.19 to 0.06)	0.33
Effect of female sex at stroke on slope, per year	-0.18 (-0.38 to 0.02)	0.09	-0.23 (-0.46 to 0.01)	0.06	-0.15 (-0.37 to 0.06)	0.15	-0.21 (-0.45 to 0.03)	0.09
Effect of post-stroke systolic BP (per 10 mm Hg increase) on slope, per year	-0.003 (-0.05 to 0.05)	0.90	NA	NA	NA	NA	0.01 (-0.06 to 0.08)	0.78
Effect of post-stroke glucose (per 10 mg/dL increase) on slope, per year	NA	NA	-0.0004 (-0.04 to 0.04)	0.99	NA	NA	0.002 (-0.04 to 0.05)	0.92
Effect of post-stroke LDL cholesterol (per 10 mg/dL increase) on slope, per year	NA	NA	NA	NA	-0.003 (-0.04 to 0.03)	0.85	-0.01 (-0.05 to 0.03)	0.53

© 2023 Levine DA et al. *JAMA Network Open.*

Abbreviations: BP=blood pressure. LDL=low-density lipoprotein. NA=not applicable.
a: All cognitive measures are set to a T-score metric (mean 50, SD 10); a 1-point difference represents a 0.1 SD difference in the distribution of cognition across the 4 cohorts. Higher cognitive scores indicate better performance.
b: Linear mixed-effects models included time since stroke, race, sex, age at time of stroke, cohort, education, income, medication for hypertension, diabetes, and high cholesterol, pre-stroke body mass index, waist circumference, smoking status, physical activity, alcohol consumption per week, history of MI, history of atrial fibrillation, glomerular filtration rate, cohort study, pre-stroke mean executive function, pre-stroke mean systolic BP, pre-stroke mean glucose, pre-stroke mean LDL, post-stroke mean systolic BP, post-stroke mean glucose, post-stroke mean LDL, age at time of stroke*time since stroke, sex*time since stroke, post-stroke mean systolic BP*time since stroke, post-stroke mean glucose*time since stroke, post-stroke mean LDL*time since stroke, anti-hypertensive medication use*time, anti-hyperglycemic medication use*time, and lipid lowering medication use*time. To consider correlation between longitudinal global cognition measures, we included random intercept and slope effect associated with participants. Glucose, LDL cholesterol, and systolic BP values are divided by 10 so that the parameter estimates refer to a 10-unit change in the variables. Each cognitive outcome is set to missing (censored) at the time of second expert-adjudicated incident stroke, death, loss to follow-up, or the end of follow-up, whichever occurs first. Models M1a, M1b, and M1c estimate the individual effect of post-stroke time-invariant mean systolic BP, glucose, and LDL cholesterol on post-stroke executive function decline with separate models. Model M1a includes a post-stroke time-invariant mean systolic BP level by time interaction and post-stroke time-invariant mean systolic BP. Model M1b includes a post-stroke time-invariant mean glucose level by time interaction and post-stroke time-invariant mean glucose. Model M1c includes post-stroke time-invariant mean LDL cholesterol level by time interaction and post-stroke time-invariant mean LDL cholesterol. Model M2 estimates the joint effect of post-stroke time-invariant mean systolic BP, glucose, and LDL cholesterol on post-stroke executive function decline. Model M2 includes the post-stroke time-invariant mean systolic BP, glucose, and LDL cholesterol and their interactions with time.
c: Median (interquartile range) number of executive function assessments before stroke was 2 (1, 4) and after stroke was 2 (1, 3).

© 2023 Levine DA et al. *JAMA Network Open.*

eTable 10. Association of Poststroke Time-Invariant Mean Vascular Risk Factor Levels and Poststroke Memory[ac] Decline Including Participants With History of Stroke at Cohort Baseline: STROKE COG Study, 1971 to 2021

	Model[b] M1a: Time-invariant post-stroke systolic BP (n=963)		Model M1b: Time-invariant post-stroke glucose (n=769)		Model M1c: Time-invariant post-stroke LDL cholesterol (n=710)		Model M2: Joint time-invariant post-stroke systolic BP, glucose, and LDL cholesterol (n=576)	
**Coefficient**	**Estimate (95% CI)**	***P***	**Estimate (95% CI)**	***P***	**Estimate (95% CI)**	***P***	**Estimate (95% CI)**	***P***
Slope (change in cognition over time), per year	-0.25 (-0.46 to -0.05)	0.02	-0.29 (-0.47 to -0.11)	0.002	-0.29 (-0.50 to -0.08)	0.007	-0.35 (-0.61 to -0.08)	0.01
Effect of age at stroke (per 10-year increase) on slope, per year	0.03 (-0.09 to 0.15)	0.64	0.04 (-0.09 to 0.17)	0.53	-0.01 (-0.15 to 0.13)	0.88	0.03 (-0.12 to 0.18)	0.64
Effect of female sex at stroke on slope, per year	-0.26 (-0.46 to -0.05)	0.02	-0.25 (-0.47 to -0.02)	0.04	-0.28 (-0.52 to -0.03)	0.03	-0.21 (-0.48 to 0.05)	0.11
Effect of post-stroke systolic BP (per 10 mm Hg increase) on slope, per year	0.03 (-0.02 to 0.09)	0.20	NA	NA	NA	NA	0.03 (-0.04 to 0.11)	0.37
Effect of post-stroke glucose (per 10 mg/dL increase) on slope, per year	NA	NA	-0.001 (-0.04 to 0.04)	0.95	NA	NA	-0.01 (-0.06 to 0.03)	0.58
Effect of post-stroke LDL cholesterol (per 10 mg/dL increase) on slope, per year	NA	NA	NA	NA	-0.003 (-0.04 to 0.03)	0.87	0.005 (-0.03 to 0.04)	0.81

© 2023 Levine DA et al. *JAMA Network Open.*

Abbreviations: BP=blood pressure. LDL=low-density lipoprotein. NA=not applicable.
a: All cognitive measures are set to a T-score metric (mean 50, SD 10); a 1-point difference represents a 0.1 SD difference in the distribution of cognition across the 4 cohorts. Higher cognitive scores indicate better performance.
b: Linear mixed-effects models included time since stroke, race, sex, age at time of stroke, cohort, education, income, medication for hypertension, diabetes, and high cholesterol, pre-stroke BMI, waist circumference, smoking status, physical activity, alcohol consumption per week, history of MI, history of atrial fibrillation, glomerular filtration rate, cohort study, pre-stroke mean memory, pre-stroke mean systolic BP, pre-stroke mean glucose, pre-stroke mean LDL, post-stroke mean systolic BP, post-stroke mean glucose, post-stroke mean LDL, age at time of stroke*time since stroke, sex*time since stroke, post-stroke mean systolic BP*time since stroke, post-stroke mean glucose*time since stroke, post-stroke mean LDL*time since stroke, anti-hypertensive medication use*time, anti-hyperglycemic medication use*time, and lipid lowering medication use*time. To consider correlation between longitudinal global cognition measures, we included random intercept and slope effect associated with participants. Glucose, LDL cholesterol, and systolic BP values are divided by 10 so that the parameter estimates refer to a 10-unit change in the variables. Each cognitive outcome is set to missing (censored) at the time of second expert-adjudicated incident stroke, death, loss to follow-up, or the end of follow-up, whichever occurs first. Models M1a, M1b, and M1c estimate the individual effect of post-stroke time-invariant mean systolic BP, glucose, and LDL cholesterol on post-stroke memory decline with separate models. Model M1a includes a post-stroke time-invariant mean systolic BP level by time interaction and post-stroke time-invariant mean systolic BP. Model M1b includes a post-stroke time-invariant mean glucose level by time interaction and post-stroke time-invariant mean glucose. Model M1c includes post-stroke time-invariant mean LDL cholesterol level by time interaction and post-stroke time-invariant mean LDL cholesterol. Model M2 estimates the joint effect of post-stroke time-invariant mean systolic BP, glucose, and LDL cholesterol on post-stroke memory decline. Model M2 includes the post-stroke time-invariant mean systolic BP, glucose, and LDL cholesterol and their interactions with time.
c: Median (interquartile range) number of memory assessments before stroke was 3 (1, 5) and after stroke was 2 (1, 3).

© 2023 Levine DA et al. *JAMA Network Open.*

eTable 11. Sensitivity Analysis of Association of Poststroke Vascular Risk Factor Levels and Post-Stroke Global Cognition[ac] Decline Requiring Participants to Have 2 or More Poststroke Cognitive Assessments: STROKE COG Study, 1971 to 2021

	Model[b] M1a: Time-varying post-stroke systolic BP (n=781)		Model M1b: Time-varying post-stroke glucose (n=707)		Model M1c: Time-varying post-stroke LDL cholesterol (n=579)		Model M2: Joint time-varying post-stroke systolic BP, glucose, and LDL cholesterol (n=544)	
**Coefficient**	**Estimate (95% CI)**	***P***	**Estimate (95% CI)**	***P***	**Estimate (95% CI)**	***P***	**Estimate (95% CI)**	***P***
Slope (change in cognition over time), per year	-0.57 (-0.76 to -0.37)	<0.001	-0.39 (-0.57 to -0.22)	<0.001	-0.50 (-0.70 to -0.31)	<0.001	-0.57 (-0.81 to -0.34)	<0.001
Effect of age at stroke (per 10-year increase) on slope, per year	-0.24 (-0.36 to -0.13)	<0.001	-0.20 (-0.32 to -0.08)	0.001	-0.19 (-0.31 to -0.07)	0.002	-0.20 (-0.33 to -0.08)	0.001
Effect of female sex at stroke on slope, per year	-0.17 (-0.37 to 0.02)	0.08	-0.26 (-0.48 to -0.05)	0.02	-0.22 (-0.44 to -0.002)	0.048	-0.29 (-0.52 to -0.06)	0.01
Effect of post-stroke systolic BP (per 10 mm Hg increase) on slope, per year	0.04 (-0.02 to 0.09)	0.19	NA	NA	NA	NA	0.03 (-0.04 to 0.10)	0.44
Effect of post-stroke glucose (per 10 mg/dL increase) on slope, per year	NA	NA	-0.02 (-0.06 to 0.02)	0.23	NA	NA	-0.04 (-0.08 to 0.00)	0.06
Effect of post-stroke LDL cholesterol (per 10 mg/dL increase) on slope, per year	NA	NA	NA	NA	0.02 (-0.01 to 0.06)	0.20	0.02 (-0.02 to 0.05)	0.36

© 2023 Levine DA et al. *JAMA Network Open.*

Abbreviations: BP=blood pressure. LDL=low-density lipoprotein. NA=not applicable.
a: Global cognitive performance measures global cognition. All cognitive measures are set to a T-score metric (mean 50, SD 10); a 1-point difference represents a 0.1 SD difference in the distribution of cognition across the 4 cohorts. Higher cognitive scores indicate better performance.
b: Linear mixed-effects models included time since stroke, race, sex, age at time of stroke, cohort, education, income, medication for hypertension, diabetes, and high cholesterol, pre-stroke body mass index, waist circumference, smoking status, physical activity, alcohol consumption per week, history of MI, history of atrial fibrillation, glomerular filtration rate, cohort study, pre-stroke mean global cognition, pre-stroke mean systolic BP, pre-stroke mean glucose, pre-stroke mean LDL, post-stroke mean systolic BP, post-stroke mean glucose, post-stroke mean LDL, age at time of stroke*time since stroke, sex*time since stroke, post-stroke mean systolic BP*time since stroke, post-stroke mean glucose*time since stroke, post-stroke mean LDL*time since stroke, anti-hypertensive medication use*time, anti-hyperglycemic medication use*time, and lipid lowering medication use*time. To consider correlation between longitudinal global cognition measures, we included random intercept and slope effect associated with participants. Glucose, LDL cholesterol, and systolic BP values are divided by 10 so that the parameter estimates refer to a 10-unit change in the variables. Each cognitive outcome is set to missing (censored) at the time of second expert-adjudicated incident stroke, death, loss to follow-up, or the end of follow-up, whichever occurs first. Models M1a, M1b, and M1c estimate the individual effect of post-stroke time-invariant mean systolic BP, glucose, and LDL cholesterol levels on global cognitive decline with separate models. Model M1a includes a post-stroke time-invariant mean systolic BP level by time interaction and post-stroke time-invariant mean systolic BP. Model M1b includes a post-stroke time-invariant mean glucose level by time interaction and post-stroke time-invariant mean glucose. Model M1c includes post-stroke time-invariant mean LDL cholesterol level by time interaction and post-stroke time-invariant mean LDL cholesterol. Model M2 estimates the joint effect of post-stroke time-invariant mean systolic BP, glucose, and LDL cholesterol) on post-stroke global cognitive decline. Model M2 includes the post-stroke time-invariant mean systolic BP, glucose, and LDL cholesterol and their interactions with time.
c: Median (interquartile range) number of global cognition assessments before stroke was 4 (2, 7) and after stroke was 3 (2, 5).

© 2023 Levine DA et al. *JAMA Network Open.*

eTable 12. Sensitivity Analysis of Association of Poststroke Vascular Risk Factor Levels With Poststroke Global Cognition[ac] Decline Including Poststroke Depressive Symptoms Among Participants With Depressive Symptom Scores: STROKE COG Study, 1971 to 2021

	Model[b] M1a: Time-varying post-stroke systolic BP (n=918)		Model M1b: Time-varying post-stroke glucose (n=767)		Model M1c: Time-varying post-stroke LDL cholesterol (n= 682)		Model M2: Joint time-varying post-stroke systolic BP, glucose, and LDL cholesterol (n= 589)	
**Coefficient**	**Estimate (95% CI)**	***P***	**Estimate (95% CI)**	***P***	**Estimate (95% CI)**	***P***	**Estimate (95% CI)**	***P***
Slope (change in cognition over time), per year	-0.38 (-0.68 to -0.08)	0.01	-0.32 (-0.64 to -0.01)	0.04	-0.43 (-0.76, to -0.10)	0.01	-0.41 (-0.76 to -0.07)	0.02
Effect of age at stroke (per 10-year increase) on slope, per year	-0.21 (-0.33 to -0.10)	<0.001	-0.19 (-0.31 to -0.07)	0.002	-0.19 (-0.31, -0.08)	0.001	-0.18 (-0.31 to -0.06)	0.005
Effect of female sex at stroke on slope, per year	-0.21 (-0.40 to -0.01)	0.04	-0.28 (-0.49 to -0.06)	0.01	-0.22 (-0.44 to -0.001)	0.049	-0.25 (-0.49 to, -0.02)	0.03
Effect of post-stroke depressive symptoms (per 1 unit increase) on slope, per year	-0.01 (-0.20 to 0.18)	0.91	-0.04 (-0.25 to 0.17)	0.71	0.03 (-0.18 to 0.25)	0.76	-0.003 (-0.24 to 0.23)	0.97
Effect of post-stroke systolic BP (per 10 mm Hg increase) on slope, per year	0.03 (-0.02 to 0.08)	0.17	NA	NA	NA	NA	0.06 (-0.01 to 0.13)	0.11
Effect of post-stroke glucose (per 10 mg/dL increase) on slope, per year	NA	NA	-0.02 (-0.06 to 0.01)	0.18	NA	NA	-0.05 (-0.09 to -0.005)	0.03
Effect of post-stroke LDL cholesterol (per 10 mg/dL increase) on slope, per year	NA	NA	NA	NA	0.01 (-0.03 to 0.04)	0.75	0.01 (-0.03 to 0.04)	0.65

© 2023 Levine DA et al. *JAMA Network Open.*

Abbreviations: BP=blood pressure. LDL=low-density lipoprotein. NA=not applicable.
a: Global cognitive performance measures global cognition. All cognitive measures are set to a T-score metric (mean 50, SD 10); a 1-point difference represents a 0.1 SD difference in the distribution of cognition across the 4 cohorts. Higher cognitive scores indicate better performance.
b: Linear mixed-effects models included depressive symptoms (CES-D score), time since stroke, race, sex, age at time of stroke, cohort, education, income, medication for hypertension, diabetes, and high cholesterol, pre-stroke body mass index, waist circumference, smoking status, physical activity, alcohol consumption per week, history of MI, history of atrial fibrillation, glomerular filtration rate, cohort study, pre-stroke mean global cognition, pre-stroke mean systolic BP, pre-stroke mean glucose, pre-stroke mean LDL, post-stroke mean systolic BP, post-stroke mean glucose, post-stroke mean LDL, age at time of stroke*time since stroke, sex*time since stroke, post-stroke mean systolic BP*time since stroke, post-stroke mean glucose*time since stroke, post-stroke mean LDL*time since stroke, anti-hypertensive medication use*time, anti-hyperglycemic medication use*time, and lipid lowering medication use*time. To consider correlation between longitudinal global cognition measures, we included random intercept and slope effect associated with participants. Glucose, LDL cholesterol, and systolic BP values are divided by 10 so that the parameter estimates refer to a 10-unit change in the variables. Each cognitive outcome is set to missing (censored) at the time of second expert-adjudicated incident stroke, death, loss to follow-up, or the end of follow-up, whichever occurs first. Models M1a, M1b, and M1c estimate the individual effect of post-stroke time-varying mean systolic BP, glucose, and LDL cholesterol on post-stroke executive function decline with separate models. Model M1a includes a post-stroke time-varying mean systolic BP level by time interaction and post-stroke time-varying mean systolic BP. Model M1b includes a post-stroke time-varying mean glucose level by time interaction and post-stroke time-varying mean glucose. Model M1c includes post-stroke time-varying mean LDL cholesterol level by time interaction and post-stroke time-varying mean LDL cholesterol. Model M2 estimates the joint effect of post-stroke time-varying mean systolic BP, glucose, and LDL cholesterol on post-stroke executive function decline. Model M2 includes the post-stroke time-varying mean systolic BP, glucose, and LDL cholesterol and their interactions with time.
c: Median (interquartile range) number of executive function assessments before stroke was 4 (2, 7) and after stroke was 3 (2, 5).

© 2023 Levine DA et al. *JAMA Network Open.*

eTable 13. Sensitivity Analysis of Association of Poststroke Vascular Risk Factor Levels With Poststroke Executive Function[ac] Decline Including Poststroke Depressive Symptoms Among Participants With Depressive Symptom Scores: STROKE COG Study, 1971 to 2021

	Model[b] M1a: Time-varying post-stroke systolic BP (n=790)		Model M1b: Time-varying post-stroke glucose (n=647)		Model M1c: Time-varying post-stroke LDL cholesterol (n=558)		Model M2: Joint time-varying post-stroke systolic BP, glucose, and LDL cholesterol (n=475)	
**Coefficient**	**Estimate (95% CI)**	***P***	**Estimate (95% CI)**	***P***	**Estimate (95% CI)**	***P***	**Estimate (95% CI)**	***P***
Slope (change in cognition over time), per year	-0.44 (-0.85 to -0.04)	0.03	-0.52 (-0.95 to, -0.10)	0.02	-0.57 (-0.97 to -0.16)	0.006	-0.53 (-0.96 to -0.10)	0.02
Effect of age at stroke (per 10-year increase) on slope, per year	-0.06 (-0.18 to 0.06)	0.31	-0.04 (-0.17 to 0.09)	0.54	-0.09 (-0.20 to 0.03)	0.16	-0.07 (-0.20 to 0.06)	0.30
Effect of female sex at stroke on slope, per year	-0.13 (-0.34 to 0.08)	0.23	-0.20 (-0.43 to 0.04)	0.10	-0.11 (-0.33 to 0.12)	0.34	-0.14 (-0.39 to 0.11)	0.28
Effect of post-stroke depressive symptoms (per 1 unit increase) on slope, per year	0.08 (-0.18 to 0.33)	0.55	0.14 (-0.12 to 0.41)	0.29	0.18 (-0.08 to 0.43)	0.18	0.14 (-0.14 to 0.42)	0.32
Effect of post-stroke systolic BP (per 10 mm Hg increase) on slope, per year	0.003 (-0.05 to 0.06)	0.91	NA	NA	NA	NA	0.02 (-0.06 to 0.09)	0.64
Effect of post-stroke glucose (per 10 mg/dL increase) on slope, per year	NA	NA	-0.0001 (-0.04 to 0.04)	0.995	NA	NA	-0.01 (-0.05 to 0.04)	0.69
Effect of post-stroke LDL cholesterol (per 10 mg/dL increase) on slope, per year	NA	NA	NA	NA	-0.02 (-0.05 to 0.01)	0.28	-0.03 (-0.06 to 0.01)	0.15

© 2023 Levine DA et al. *JAMA Network Open.*

Abbreviations: BP=blood pressure. LDL=low-density lipoprotein. NA=not applicable.
a: All cognitive measures are set to a T-score metric (mean 50, SD 10); a 1-point difference represents a 0.1 SD difference in the distribution of cognition across the 4 cohorts. Higher cognitive scores indicate better performance.
b: Linear mixed-effects models included depressive symptoms (CES-D score), time since stroke, race, sex, age at time of stroke, cohort, education, income, medication for hypertension, diabetes, and high cholesterol, pre-stroke body mass index, waist circumference, smoking status, physical activity, alcohol consumption per week, history of MI, history of atrial fibrillation, glomerular filtration rate, cohort study, pre-stroke mean executive function, pre-stroke mean systolic BP, pre-stroke mean glucose, pre-stroke mean LDL, post-stroke mean systolic BP, post-stroke mean glucose, post-stroke mean LDL, age at time of stroke*time since stroke, sex*time since stroke, post-stroke mean systolic BP*time since stroke, post-stroke mean glucose*time since stroke, post-stroke mean LDL*time since stroke, anti-hypertensive medication use*time, anti-hyperglycemic medication use*time, and lipid lowering medication use*time. To consider correlation between longitudinal global cognition measures, we included random intercept and slope effect associated with participants. Glucose, LDL cholesterol, and systolic BP values are divided by 10 so that the parameter estimates refer to a 10-unit change in the variables. Each cognitive outcome is set to missing (censored) at the time of second expert-adjudicated incident stroke, death, loss to follow-up, or the end of follow-up, whichever occurs first. Models M1a, M1b, and M1c estimate the individual effect of post-stroke time-varying mean systolic BP, glucose, and LDL cholesterol on post-stroke executive function decline with separate models. Model M1a includes a post-stroke time-varying mean systolic BP level by time interaction and post-stroke time-varying mean systolic BP. Model M1b includes a post-stroke time-varying mean glucose level by time interaction and post-stroke time-varying mean glucose. Model M1c includes post-stroke time-varying mean LDL cholesterol level by time interaction and post-stroke time-varying mean LDL cholesterol. Model M2 estimates the joint effect of post-stroke time-varying mean systolic BP, glucose, and LDL cholesterol on post-stroke executive function decline. Model M2 includes the post-stroke time-varying mean systolic BP, glucose, and LDL cholesterol and their interactions with time.
c: Median (interquartile range) number of executive function assessments before stroke was 3 (2, 4) and after stroke was 2 (1, 3).

© 2023 Levine DA et al. *JAMA Network Open.*

eTable 14. Sensitivity Analysis of Association of Poststroke Vascular Risk Factor Levels With Poststroke Memory[ac] Decline Including Poststroke Depressive Symptoms Among Participants With Depressive Symptom Scores: STROKE COG Study, 1971 to 2019

	Model[b] M1a: Time-varying post-stroke systolic BP (n=869)		Model M1b: Time-varying post-stroke glucose (n=719)		Model M1c: Time-varying post-stroke LDL cholesterol (n=623)		Model M2: Joint time-varying post-stroke systolic BP, glucose, and LDL cholesterol (n=530)	
**Coefficient**	**Estimate (95% CI)**	***P***	**Estimate (95% CI)**	***P***	**Estimate (95% CI)**	***P***	**Estimate (95% CI)**	***P***
Slope (change in cognition over time), per year	-0.31 (-0.62 to, 0.01)	0.05	-0.24 (-0.55 to 0.08)	0.14	-0.39 (-0.75 to -0.03)	0.04	-0.41 (-0.79 to -0.03)	0.03
Effect of age at stroke (per 10-year increase) on slope, per year	0.01 (-0.11 to 0.13)	0.91	0.03 (-0.10 to 0.16)	0.67	-0.04 (-0.17 to 0.10)	0.61	-0.004 (-0.15 to 0.15)	0.96
Effect of female sex at stroke on slope, per year	-0.29 (-0.51 to -0.08)	0.008	-0.28 (-0.52 to -0.05)	0.02	-0.34 (-0.59 to -0.09)	0.008	-0.28 (-0.55 to -0.01)	0.04
Effect of post-stroke depressive symptoms (per 1 unit increase) on slope, per year	0.02 (-0.19 to 0.22)	0.88	-0.01 (-0.23 to 0.20)	0.89	0.06 (-0.18 to 0.31)	0.60	0.05 (-0.21 to 0.31)	0.71
Effect of post-stroke systolic BP (per 10 mm Hg increase) on slope, per year	0.03 (-0.03 to 0.08)	0.33	NA	NA	NA	NA	0.04 (-0.04 to 0.12)	0.33
Effect of post-stroke glucose (per 10 mg/dL increase) on slope, per year	NA	NA	-0.01 (-0.05 to 0.02)	0.45	NA	NA	-0.03 (-0.08 to 0.01)	0.16
Effect of post-stroke LDL cholesterol (per 10 mg/dL increase) on slope, per year	NA	NA	NA	NA	-0.002 (-0.04 to 0.03)	0.93	0.01 (-0.03 to 0.05)	0.79

© 2023 Levine DA et al. *JAMA Network Open.*

Abbreviations: BP=blood pressure. LDL=low-density lipoprotein. NA=not applicable.
a: All cognitive measures are set to a T-score metric (mean 50, SD 10); a 1-point difference represents a 0.1 SD difference in the distribution of cognition across the 4 cohorts. Higher cognitive scores indicate better performance.
b: Linear mixed-effects models included depressive symptoms (CES-D score), time since stroke, race, sex, age at time of stroke, cohort, education, income, medication for hypertension, diabetes, and high cholesterol, pre-stroke BMI, waist circumference, smoking status, physical activity, alcohol consumption per week, history of MI, history of atrial fibrillation, glomerular filtration rate, cohort study, pre-stroke mean memory, pre-stroke mean systolic BP, pre-stroke mean glucose, pre-stroke mean LDL, post-stroke mean systolic BP, post-stroke mean glucose, post-stroke mean LDL, age at time of stroke*time since stroke, sex*time since stroke, post-stroke mean systolic BP*time since stroke, post-stroke mean glucose*time since stroke, post-stroke mean LDL*time since stroke, anti-hypertensive medication use*time, anti-hyperglycemic medication use*time, and lipid lowering medication use*time. To consider correlation between longitudinal global cognition measures, we included random intercept and slope effect associated with participants. Glucose, LDL cholesterol, and systolic BP values are divided by 10 so that the parameter estimates refer to a 10-unit change in the variables. Each cognitive outcome is set to missing (censored) at the time of second expert-adjudicated incident stroke, death, loss to follow-up, or the end of follow-up, whichever occurs first. Models M1a, M1b, and M1c estimate the individual effect of post-stroke time-varying mean systolic BP, glucose, and LDL cholesterol on post-stroke memory decline with separate models. Model M1a includes a post-stroke time-varying mean systolic BP level by time interaction and post-stroke time-varying mean systolic BP. Model M1b includes a post-stroke time-varying mean glucose level by time interaction and post-stroke time-varying mean glucose. Model M1c includes post-stroke time-varying mean LDL cholesterol level by time interaction and post-stroke time-varying mean LDL cholesterol. Model M2 estimates the joint effect of post-stroke time-varying mean systolic BP, glucose, and LDL cholesterol on post-stroke memory decline. Model M2 includes the post-stroke time-varying mean systolic BP, glucose, and LDL cholesterol and their interactions with time.
c: Median (interquartile range) number of memory assessments before stroke was 3 (2, 6) and after stroke was 2 (1, 3).

© 2023 Levine DA et al. *JAMA Network Open.*

eTable 15. Sensitivity Analysis of Association of Poststroke Vascular Risk Factor Levels With Poststroke Global Cognition[a] Decline by Cohort: STROKE COG Study, 1971 to 2021

ARIC Cohort								
	Model[b] M1a: Time-varying post-stroke systolic BP (n= 238)		Model M1b: Time-varying post-stroke glucose (n= 127)		Model M1c: Time-varying post-stroke LDL cholesterol (n= 227)		Model M2: Joint time-varying post-stroke systolic BP, glucose, and LDL cholesterol (n= 126)	
**Coefficient**	**Estimate (95% CI)**	***P***	**Estimate (95% CI)**	***P***	**Estimate (95% CI)**	***P***	**Estimate (95% CI)**	***P***
Slope (change in cognition over time), per year	-0.19 (-0.48 to 0.09)	0.17	-0.14 (-0.49 to 0.20)	0.41	-0.20 (-0.50 to 0.09)	0.18	-0.26 (-0.90 to 0.38)	0.42
Effect of age at stroke (per 10-year increase) on slope, per year	-0.32 (-0.50 to -0.14)	<0.001	-0.39 (-0.66 to -0.13)	0.003	-0.44 (-0.63 to -0.26)	<0.001	-0.34 (-0.67 to -0.01)	0.04
Effect of female sex at stroke on slope, per year	-0.30 (-0.59 to -0.01)	0.04	-0.33 (-0.80 to 0.15)	0.17	-0.30 (-0.58 to -0.01)	0.04	-0.32 (-0.83 to 0.20)	0.23
Effect of post-stroke systolic BP (per 10 mm Hg increase) on slope, per year	0.06 (-0.01 to 0.14)	0.10	NA	NA	NA	NA	0.13 (-0.04 to 0.28)	0.13
Effect of post-stroke glucose (per 10 mg/dL increase) on slope, per year	NA	NA	-0.02 (-0.13 to 0.10)	0.78	NA	NA	-0.02 (-0.14 to 0.11)	0.81
Effect of post-stroke LDL cholesterol (per 10 mg/dL increase) on slope, per year	NA	NA	NA	NA	-0.04 (-0.09 to 0.01)	0.14	-0.05 (-0.14 to 0.05)	0.32
**ARIC Cohort:** Median (interquartile range) number of global cognition assessments before stroke was 2 (1, 3) and after stroke was 1 (1, 2).								

© 2023 Levine DA et al. *JAMA Network Open.*

CHS Cohort								
	**Model M1a: Time-varying post-stroke systolic BP (n= 332)**		**Model M1b: Time-varying post-stroke glucose (n= 277)**		**Model M1c: Time-varying post-stroke LDL cholesterol (n=125)**		**Model M2: Joint time-varying post-stroke systolic BP, glucose, and LDL cholesterol (n= 125)**	
**Coefficient**	**Estimate (95% CI)**	***P***	**Estimate (95% CI)**	***P***	**Estimate (95% CI)**	***P***	**Estimate (95% CI)**	***P***
Slope (change in cognition over time), per year	-0.76 (-1.32 to -0.19)	0.009	-0.60 (-1.20 to -0.004)	0.05	-0.59 (-1.24 to 0.06)	0.07	-0.25 (-0.96 to 0.46)	0.49
Effect of age at stroke (per 10-year increase) on slope, per year	-0.29 (-0.65 to 0.08)	0.13	-0.20 (-0.62 to 0.22)	0.36	0.04 (-0.43 o 0.51)	0.86	-0.12 (-0.60 to 0.35)	0.61
Effect of female sex at stroke on slope, per year	-0.23 (-0.62 to 0.15)	0.23	-0.39 (-0.84 to 0.05)	0.08	-0.61 (-1.12 to -0.11)	0.02	-0.71 (-1.22 to -0.20)	0.007
Effect of post-stroke systolic BP (per 10 mm Hg increase) on slope, per year	0.14 (0.04 to, 0.24)	0.007	NA	NA	NA	NA	0.18 (0.05 to 0.31)	0.007
Effect of post-stroke glucose (per 10 mg/dL increase) on slope, per year	NA	NA	-0.001 (-0.07 to 0.07)	0.98	NA	NA	-0.07 (-0.16 to 0.02)	0.13
Effect of post-stroke LDL cholesterol (per 10 mg/dL increase) on slope, per year	NA	NA	NA	NA	-0.003 (-0.07 to 0.07)	0.94	0.01 (-0.06 to 0.07)	0.86
**CHS Cohort:** Median (interquartile range) number of global cognition assessments before stroke was 4 (2, 7) and after stroke was 3 (2, 5).								

© 2023 Levine DA et al. *JAMA Network Open.*

FOS Cohort								
	**Model M1a: Time-varying post-stroke systolic BP (n=101)**		**Model M1b: Time-varying post-stroke glucose (n=88)**		**Model M1c: Time-varying post-stroke LDL cholesterol (n=88)**		**Model M2: Joint time-varying post-stroke systolic BP, glucose, and LDL cholesterol (n=86)**	
**Coefficient**	**Estimate (95% CI)**	***P***	**Estimate (95% CI)**	***P***	**Estimate (95% CI)**	***P***	**Estimate (95% CI)**	***P***
Slope (change in cognition over time), per year	-0.58 (-1.09 to, -0.08)	0.02	-0.27 (-0.70 to 0.15)	0.21	-0.66 (-1.15 to -0.17)	0.009	-0.80 (-1.38 to -0.22)	0.007
Effect of age at stroke (per 10-year increase) on slope, per year	-0.17 (-0.45 to 0.11)	0.24	-0.17 (-0.43 to 0.08)	0.18	-0.26 (-0.52 to -0.01)	0.045	-0.24 (-0.52 to 0.04)	0.09
Effect of female sex at stroke on slope, per year	-0.35 (-0.90 to 0.21)	0.22	-0.54 (-1.09 to 0.01)	0.05	-0.39 (-0.92 to 0.13)	0.14	-0.50 (-1.06 to 0.06)	0.08
Effect of post-stroke systolic BP (per 10 mm Hg increase) on slope, per year	-0.05 (-0.21 to 0.11)	0.55	NA	NA	NA	NA	-0.04 (-0.21 to 0.12)	0.62
Effect of post-stroke glucose (per 10 mg/dL increase) on slope, per year	NA	NA	0.06 (-0.07 to 0.18)	0.36	NA	NA	0.06 (-0.08 to 0.19)	0.39
Effect of post-stroke LDL cholesterol (per 10 mg/dL increase) on slope, per year	NA	NA	NA	NA	0.08 (-0.01 to 0.16)	0.07	0.07 (-0.01 to 0.16)	0.10
**FOS Cohort:** Median (interquartile range) number of global cognitions before stroke was 4 (2, 5) and after stroke was 2 (1, 3).								

© 2023 Levine DA et al. *JAMA Network Open.*

REGARDS Cohort								
	Model M1a: Time-varying post-stroke systolic BP (n=311)		Model M1b: Time-varying post-stroke glucose (n=295)		Model M1c: Time-varying post-stroke LDL cholesterol (n=294)		Model M2: Joint time-varying post-stroke systolic BP, glucose, and LDL cholesterol (n=272)	
**Coefficient**	**Estimate (95% CI)**	***P***	**Estimate (95% CI)**	***P***	**Estimate (95% CI)**	***P***	**Estimate (95% CI)**	***P***
Slope (change in cognition over time), per year	-0.37 (-0.77 to 0.03)	0.07	-0.33 (-0.68 to 0.01)	0.06	-0.26 (-0.64 to 0.12)	0.18	-0.40 (-0.85 to 0.05)	0.08
Effect of age at stroke (per 10-year increase) on slope, per year	-0.18 (-0.45 to 0.10)	0.20	-0.17 (-0.45 to 0.10)	0.22	-0.19 (-0.47 to 0.09)	0.19	-0.16 (-0.45 to 0.14)	0.29
Effect of female sex at stroke on slope, per year	-0.18 (-0.60 to 0.24)	0.41	-0.20 (-0.64 to 0.23)	0.36	-0.21 (-0.66 to 0.22)	0.33	-0.24 (-0.71 to 0.22)	0.31
Effect of post-stroke systolic BP (per 10 mm Hg increase) on slope, per year	-0.06 (-0.20 to 0.07)	0.34	NA	NA	NA	NA	-0.04 (-0.18 to 0.11)	0.61
Effect of post-stroke glucose (per 10 mg/dL increase) on slope, per year	NA	NA	-0.04 (-0.11 to 0.02)	0.16	NA	NA	-0.06 (-0.13 to 0.02)	0.13
Effect of post-stroke LDL cholesterol (per 10 mg/dL increase) on slope, per year	NA	NA	NA	NA	0.03 (-0.04 to 0.10)	0.40	0.04 (-0.03 to 0.11)	0.27
**REGARDS Cohort:** Median (interquartile range) number of global cognition assessments before stroke was 6 (4, 9) and after stroke was 4 (3, 5).								

© 2023 Levine DA et al. *JAMA Network Open.*

Abbreviations: BP=blood pressure. LDL=low-density lipoprotein. NA=not applicable.
a: All cognitive measures are set to a T-score metric (mean 50, SD 10); a 1-point difference represents a 0.1 SD difference in the distribution of cognition across the 4 cohorts. Higher cognitive scores indicate better performance.
b: Linear mixed-effects models included time since stroke, race, sex, age at time of stroke, education, income, medication for hypertension, diabetes, and high cholesterol, pre-stroke body mass index, waist circumference, smoking status, physical activity, alcohol consumption per week, history of MI, history of atrial fibrillation, glomerular filtration rate, cohort study, pre-stroke mean global cognition, pre-stroke mean systolic BP, pre-stroke mean glucose, pre-stroke mean LDL, post-stroke mean systolic BP, post-stroke mean glucose, post-stroke mean LDL, age at time of stroke*time since stroke, sex*time since stroke, post-stroke mean systolic BP*time since stroke, post-stroke mean glucose*time since stroke, post-stroke mean LDL*time since stroke, anti-hypertensive medication use*time, anti-hyperglycemic medication use*time, and lipid lowering medication use*time. To consider correlation between longitudinal global cognition measures, we included random intercept and slope effect associated with participants. Glucose, LDL cholesterol, and systolic BP values are divided by 10 so that the parameter estimates refer to a 10-unit change in the variables. Each cognitive outcome is set to missing (censored) at the time of second expert-adjudicated incident stroke, death, loss to follow-up, or the end of follow-up, whichever occurs first. Models M1a, M1b, and M1c estimate the individual effect of post-stroke time-varying mean systolic BP, glucose, and LDL cholesterol on post-stroke executive function decline with separate models. Model M1a includes a post-stroke time-varying mean systolic BP level by time interaction and post-stroke time-varying mean systolic BP. Model M1b includes a post-stroke time-varying mean glucose level by time interaction and post-stroke time-varying mean glucose. Model M1c includes post-stroke time-varying mean LDL cholesterol level by time interaction and post-stroke time-varying mean LDL cholesterol. Model M2 estimates the joint effect of post-stroke time-varying mean systolic BP, glucose, and LDL cholesterol on post-stroke executive function decline. Model M2 includes the post-stroke time-varying mean systolic BP, glucose, and LDL cholesterol and their interactions with time.

© 2023 Levine DA et al. *JAMA Network Open.*

eTable 16. Sensitivity Analysis of Association of Poststroke Vascular Risk Factor Levels With Post-Stroke Executive Function[a] Decline by Cohort: STROKE COG Study, 1971 to 2019

ARIC Cohort								
	Model[b] M1a: Time-varying post-stroke systolic BP (n=236)		Model M1b: Time-varying post-stroke glucose (n=127)		Model M1c: Time-varying post-stroke LDL cholesterol (n=225)		Model M2: Joint time-varying post-stroke systolic BP, glucose, and LDL cholesterol (n=126)	
**Coefficient**	**Estimate (95% CI)**	***P***	**Estimate (95% CI)**	***P***	**Estimate (95% CI)**	***P***	**Estimate (95% CI)**	***P***
Slope (change in cognition over time), per year	-0.35 (-0.68 to -0.03)	0.03	-0.27 (-0.61 to 0.06)	0.11	-0.37 (-0.70 to -0.03)	0.03	-0.57 (-1.19 to 0.06)	0.07
Effect of age at stroke (per 10-year increase) on slope, per year	-0.19 (-0.39 to 0.01)	0.07	-0.22 (-0.50 to 0.06)	0.12	-0.20 (-0.42 to 0.02)	0.07	-0.25 (-0.58 to 0.08)	0.14
Effect of female sex at stroke on slope, per year	-0.21 (-0.53 to 0.12)	0.21	-0.02 (-0.49 to 0.45)	0.93	-0.15 (-0.48 to 0.19)	0.39	0.01 (-0.49 to 0.52)	0.95
Effect of post-stroke systolic BP (per 10 mm Hg increase) on slope, per year	-0.03 (-0.11 to 0.05)	0.45	NA	NA	NA	NA	0.01 (-0.15 to 0.16)	0.95
Effect of post-stroke glucose (per 10 mg/dL increase) on slope, per year	NA	NA	0.004 (-0.12 to 0.12)	0.95	NA	NA	-0.01 (-0.13 to 0.12)	0.93
Effect of post-stroke LDL cholesterol (per 10 mg/dL increase) on slope, per year	NA	NA	NA	NA	0.01 (-0.04 to 0.07)	0.61	0.01 (-0.08 to 0.10)	0.84
**ARIC Cohort:** Median (interquartile range) number of executive function assessments before stroke was 2 (1, 3) and after stroke was 1 (1, 2).								

© 2023 Levine DA et al. *JAMA Network Open.*

CHS Cohort								
	**Model M1a: Time-varying post-stroke systolic BP (n=332)**		**Model M1b: Time-varying post-stroke glucose (n=277)**		**Model M1c: Time-varying post-stroke LDL cholesterol (n=125)**		**Model M2: Joint time-varying post-stroke systolic BP, glucose, and LDL cholesterol (n=125)**	
**Coefficient**	**Estimate (95% CI)**	***P***	**Estimate (95% CI)**	***P***	**Estimate (95% CI)**	***P***	**Estimate (95% CI)**	***P***
Slope (change in cognition over time), per year	-0.71 (-1.34 to -0.09)	0.02	-0.72 (-1.31 to -0.14)	0.02	-0.58 (-1.20 to 0.03)	0.06	-0.61 (-1.40 to 0.18)	0.13
Effect of age at stroke (per 10-year increase) on slope, per year	0.15 (-0.25 to 0.54)	0.47	0.29 (-0.12 to 0.71)	0.16	0.43 (-0.01 to 0.88)	0.06	0.47 (-0.05 to 0.99)	0.07
Effect of female sex at stroke on slope, per year	-0.11 (-0.53 to 0.31)	0.60	-0.25 (-0.69 to 0.18)	0.26	-0.29 (-0.77 to 0.19)	0.23	-0.27 (-0.81 to 0.28)	0.34
Effect of post-stroke systolic BP (per 10 mm Hg increase) on slope, per year	0.08 (-0.03 to 0.19)	0.16	NA	NA	NA	NA	0.09 (-0.04 to 0.23)	0.17
Effect of post-stroke glucose (per 10 mg/dL increase) on slope, per year	NA	NA	-0.04 (-0.11 to 0.03)	0.31	NA	NA	-0.02 (-0.12 to 0.08)	0.67
Effect of post-stroke LDL cholesterol (per 10 mg/dL increase) on slope, per year	NA	NA	NA	NA	-0.06 (-0.12 to 0.01)	0.09	-0.05 (-0.12 to 0.02)	0.15
**CHS Cohort:** Median (interquartile range) number of executive function assessments before stroke was 4 (2, 7) and after stroke was 3 (2, 5).								

© 2023 Levine DA et al. *JAMA Network Open.*

FOS Cohort								
	**Model M1a: Time-varying post-stroke systolic BP (n=101)**		**Model M1b: Time-varying post-stroke glucose (n=88)**		**Model M1c: Time-varying post-stroke LDL cholesterol (n=88)**		**Model M2: Joint time-varying post-stroke systolic BP, glucose, and LDL cholesterol (n=86)**	
**Coefficient**	**Estimate (95% CI)**	***P***	**Estimate (95% CI)**	***P***	**Estimate (95% CI)**	***P***	**Estimate (95% CI)**	***P***
Slope (change in cognition over time), per year	-0.60 (-1.03 to -0.17)	0.007	-0.23 (-0.63 to 0.17)	0.25	-0.47 (-0.93 to -0.003)	0.048	-0.56 (-1.10 to -0.01)	0.045
Effect of age at stroke (per 10-year increase) on slope, per year	-0.19 (-0.44 to 0.05)	0.12	-0.16 (-0.39 to 0.07)	0.17	-0.20 (-0.44 to 0.03)	0.09	-0.20 (-0.45 to 0.06)	0.13
Effect of female sex at stroke on slope, per year	-0.22 (-0.70 to 0.25)	0.35	-0.37 (-0.87 to 0.13)	0.15	-0.31 (-0.79 to 0.17)	0.20	-0.43 (-0.95 to 0.08)	0.10
Effect of post-stroke systolic BP (per 10 mm Hg increase) on slope, per year	-0.02 (-0.17 to 0.14)	0.83	NA	NA	NA	NA	-0.04 (-0.21 to 0.13)	0.64
Effect of post-stroke glucose (per 10 mg/dL increase) on slope, per year	NA	NA	0.02 (-0.10 to 0.15)	0.70	NA	NA	-0.02 (-0.16 to 0.12)	0.79
Effect of post-stroke LDL cholesterol (per 10 mg/dL increase) on slope, per year	NA	NA	NA	NA	0.01 (-0.07 to 0.08)	0.88	0.002 (-0.08 to 0.08)	0.96
**FOS Cohort:** Median (interquartile range) number of executive function assessments before stroke was 4 (2, 5) and after stroke was 2 (1, 3)								

© 2023 Levine DA et al. *JAMA Network Open.*

REGARDS Cohort								
	Model M1a: Time-varying post-stroke systolic BP (n=184)		Model M1b: Time-varying post-stroke glucose (n=175)		Model M1c: Time-varying post-stroke LDL cholesterol (n=171)		Model M2: Joint time-varying post-stroke systolic BP, glucose, and LDL cholesterol (n=158)	
**Coefficient**	**Estimate (95% CI)**	***P***	**Estimate (95% CI)**	***P***	**Estimate (95% CI)**	***P***	**Estimate (95% CI)**	***P***
Slope (change in cognition over time), per year	-0.99 (-2.08 to 0.09)	0.07	-0.89 (-1.78 to 0.01)	0.05	-0.82 (-1.78 to 0.14)	0.09	-1.00 (-2.26 to 0.26)	0.12
Effect of age at stroke (per 10-year increase) on slope, per year	0.06 (-0.62 to 0.74)	0.90	-0.10 (-0.78 to 0.58)	0.77	-0.24 (-0.92 to 0.44)	0.48	0.19 (-0.59 to 0.98)	0.63
Effect of female sex at stroke on slope, per year	-0.32 (-1.27 to 0.62)	0.50	-0.06 (-1.03 to 0.92)	0.91	-0.20 (-1.21 to 0.81)	0.70	-0.37 (-1.44 to 0.70)	0.50
Effect of post-stroke systolic BP (per 10 mm Hg increase) on slope, per year	0.15 (-0.15 to 0.45)	0.34	NA	NA	NA	NA	0.17 (-0.17 to 0.52)	0.33
Effect of post-stroke glucose (per 10 mg/dL increase) on slope, per year	NA	NA	-0.01 (-0.16 to 0.14)	0.90	NA	NA	0.08 (-0.10 to 0.26)	0.39
Effect of post-stroke LDL cholesterol (per 10 mg/dL increase) on slope, per year	NA	NA	NA	NA	0.02 (-0.11 to 0.15)	0.78	-0.01 (-0.16 to 0.13)	0.84
**REGARDS Cohort:** Median (interquartile range) number of executive function assessments before stroke was 2 (1, 3) and after stroke was 1 (1, 2).								

© 2023 Levine DA et al. *JAMA Network Open.*

Abbreviations: BP=blood pressure. LDL=low-density lipoprotein. NA=not applicable.
a: All cognitive measures are set to a T-score metric (mean 50, SD 10); a 1-point difference represents a 0.1 SD difference in the distribution of cognition across the 4 cohorts. Higher cognitive scores indicate better performance.
b: Linear mixed-effects models included time since stroke, race, sex, age at time of stroke, education, income, medication for hypertension, diabetes, and high cholesterol, pre-stroke body mass index, waist circumference, smoking status, physical activity, alcohol consumption per week, history of MI, history of atrial fibrillation, glomerular filtration rate, cohort study, pre-stroke mean executive function, pre-stroke mean systolic BP, pre-stroke mean glucose, pre-stroke mean LDL, post-stroke mean systolic BP, post-stroke mean glucose, post-stroke mean LDL, age at time of stroke*time since stroke, sex*time since stroke, post-stroke mean systolic BP*time since stroke, post-stroke mean glucose*time since stroke, post-stroke mean LDL*time since stroke, anti-hypertensive medication use*time, anti-hyperglycemic medication use*time, and lipid lowering medication use*time. To consider correlation between longitudinal global cognition measures, we included random intercept and slope effect associated with participants. Glucose, LDL cholesterol, and systolic BP values are divided by 10 so that the parameter estimates refer to a 10-unit change in the variables. Each cognitive outcome is set to missing (censored) at the time of second expert-adjudicated incident stroke, death, loss to follow-up, or the end of follow-up, whichever occurs first. Models M1a, M1b, and M1c estimate the individual effect of post-stroke time-varying mean systolic BP, glucose, and LDL cholesterol on post-stroke memory decline with separate models. Model M1a includes a post-stroke time-varying mean systolic BP level by time interaction and post-stroke time-varying mean systolic BP. Model M1b includes a post-stroke time-varying mean glucose level by time interaction and post-stroke time-varying mean glucose. Model M1c includes post-stroke time-varying mean LDL cholesterol level by time interaction and post-stroke time-varying mean LDL cholesterol. Model M2 estimates the joint effect of post-stroke time-varying mean systolic BP, glucose, and LDL cholesterol on post-stroke memory decline. Model M2 includes the post-stroke time-varying mean systolic BP, glucose, and LDL cholesterol and their interactions with time.

© 2023 Levine DA et al. *JAMA Network Open.*

eTable 17. Sensitivity Analysis of Association of Poststroke Vascular Risk Factor Levels With Poststroke Memory[a] Decline by Cohort: STROKE COG Study, 1971 to 2019

ARIC Cohort								
	Model[b] M1a: Time-varying post-stroke systolic BP (n=236)		Model M1b: Time-varying post-stroke glucose (n=125)		Model M1c: Time-varying post-stroke LDL cholesterol (n=225)		Model M2: Joint time-varying post-stroke systolic BP, glucose, and LDL cholesterol (n=124)	
**Coefficient**	**Estimate (95% CI)**	***P***	**Estimate (95% CI)**	***P***	**Estimate (95% CI)**	***P***	**Estimate (95% CI)**	***P***
Slope (change in cognition over time), per year	-0.03 (-0.42 to 0.36)	0.88	-0.21 (-0.68 to 0.26)	0.38	-0.17 (-0.60 to 0.26)	0.43	-0.25 (-1.14 to 0.63)	0.57
Effect of age at stroke (per 10-year increase) on slope, per year	-0.06 (-0.31 to 0.19)	0.65	-0.18 (-0.54 to 0.18)	0.33	-0.21 (-0.48 to 0.07)	0.14	-0.06 (-0.51 to 0.39)	0.80
Effect of female sex at stroke on slope, per year	-0.66 (-1.07 to -0.26)	0.001	-0.58 (-1.20 to 0.04)	0.07	-0.62 (-1.04 to -0.21)	0.004	-0.62 (-1.30 to 0.07)	0.08
Effect of post-stroke systolic BP (per 10 mm Hg increase) on slope, per year	0.02 (-0.09 to 0.13)	0.72	NA	NA	NA	NA	0.19 (-0.04 to 0.43)	0.11
Effect of post-stroke glucose (per 10 mg/dL increase) on slope, per year	NA	NA	0.01 (-0.15 to 0.18)	0.87	NA	NA	0.04 (-0.14 to 0.22)	0.62
Effect of post-stroke LDL cholesterol (per 10 mg/dL increase) on slope, per year	NA	NA	NA	NA	-0.07 (-0.14 to 0.01)	0.07	-0.10 (-0.24 to 0.04)	0.17
**ARIC Cohort:** Median (interquartile range) number of memory function assessments before stroke was 2 (1, 2) and after stroke was 1 (1, 2).								

© 2023 Levine DA et al. *JAMA Network Open.*

CHS Cohort								
	**Model M1a: Time-varying post-stroke systolic BP (n=288)**		**Model M1b: Time-varying post-stroke glucose (n=235)**		**Model M1c: Time-varying post-stroke LDL cholesterol (n=72)**		**Model M2: Joint time-varying post-stroke systolic BP, glucose, and LDL cholesterol (n=72)**	
**Coefficient**	**Estimate (95% CI)**	***P***	**Estimate (95% CI)**	***P***	**Estimate (95% CI)**	***P***	**Estimate (95% CI)**	***P***
Slope (change in cognition over time), per year	0.14 (-0.40 to 0.67)	0.61	0.09 (-0.45 to 0.63)	0.76	0.01 (-0.72 to 0.75)	0.97	0.34 (-0.51 to 1.19)	0.43
Effect of age at stroke (per 10-year increase) on slope, per year	-0.16 (-0.52 to 0.20)	0.39	-0.05 (-0.45 to 0.35)	0.82	0.05 (-0.57 to 0.66)	0.88	0.07 (-0.62 to 0.76)	0.85
Effect of female sex at stroke on slope, per year	-0.20 (-0.59, 0.19)	0.32	-0.29 (-0.72 to 0.15)	0.19	0.003 (-0.57 to 0.58)	0.99	0.02 (-0.63 to 0.68)	0.94
Effect of post-stroke systolic BP (per 10 mm Hg increase) on slope, per year	0.11 (0.004 to 0.22)	0.04	NA	NA	NA	NA	0.08 (-0.11 to 0.26)	0.42
Effect of post-stroke glucose (per 10 mg/dL increase) on slope, per year	NA	NA	0.01 (-0.06 to 0.07)	0.87	NA	NA	0.03 (-0.08 to 0.13)	0.61
Effect of post-stroke LDL cholesterol (per 10 mg/dL increase) on slope, per year	NA	NA	NA	NA	-0.03 (-0.12 to 0.06)	0.51	-0.04 (-0.14 to 0.05)	0.39
**CHS Cohort:** Median (interquartile range) number of memory function assessments before stroke was 4 (2, 6) and after stroke was 3 (1, 4).								

© 2023 Levine DA et al. *JAMA Network Open.*

FOS Cohort								
	**Model M1a: Time-varying post-stroke systolic BP (n=100)**		**Model M1b: Time-varying post-stroke glucose (n=88)**		**Model M1c: Time-varying post-stroke LDL cholesterol (n=88)**		**Model M2: Joint time-varying post-stroke systolic BP, glucose, and LDL cholesterol (n=86)**	
**Coefficient**	**Estimate (95% CI)**	***P***	**Estimate (95% CI)**	***P***	**Estimate (95% CI)**	***P***	**Estimate (95% CI)**	***P***
Slope (change in cognition over time), per year	-0.79 (-1.33 to -0.25)	0.004	-0.47 (-0.95 to 0.01)	0.05	-0.78 (-1.33 to -0.24)	0.005	-1.02 (-1.68 to -0.36)	0.003
Effect of age at stroke (per 10-year increase) on slope, per year	-0.33 (-0.64 to -0.03)	0.03	-0.26 (-0.54 to 0.01)	0.06	-0.32 (-0.60 to -0.05)	0.02	-0.35 (-0.66 to -0.04)	0.03
Effect of female sex at stroke on slope, per year	-0.24 (-0.83 to 0.36)	0.43	-0.51 (-1.11 to 0.10)	0.10	-0.45 (-1.01 to 0.12)	0.12	-0.41 (-1.04 to 0.22)	0.20
Effect of post-stroke systolic BP (per 10 mm Hg increase) on slope, per year	0.08 (-0.11 to 0.27)	0.39	NA	NA	NA	NA	0.04 (-0.16 to 0.24)	0.72
Effect of post-stroke glucose (per 10 mg/dL increase) on slope, per year	NA	NA	-0.01 (-0.16 to 0.14)	0.88	NA	NA	-0.03 (-0.20 to 0.14)	0.73
Effect of post-stroke LDL cholesterol (per 10 mg/dL increase) on slope, per year	NA	NA	NA	NA	0.08 (-0.01 to 0.18)	0.07	0.09 (-0.01 to 0.19)	0.08
**FOS Cohort:** Median (interquartile range) number of memory assessments before stroke was 4 (2, 5) and after stroke was 2 (1, 4).								

© 2023 Levine DA et al. *JAMA Network Open.*

REGARDS Cohort								
	Model M1a: Time-varying post-stroke systolic BP (n=305)		Model M1b: Time-varying post-stroke glucose (n=289)		Model M1c: Time-varying post-stroke LDL cholesterol (n=288)		Model M2: Joint time-varying post-stroke systolic BP, glucose, and LDL cholesterol (n=266)	
**Coefficient**	**Estimate (95% CI)**	***P***	**Estimate (95% CI)**	***P***	**Estimate (95% CI)**	***P***	**Estimate (95% CI)**	***P***
Slope (change in cognition over time), per year	-0.10 (-0.36 to 0.16)	0.44	-0.18 (-0.40 to 0.05)	0.13	-0.05 (-0.31 to 0.20)	0.67	-0.11 (-0.41 to 0.18)	0.44
Effect of age at stroke (per 10-year increase) on slope, per year	-0.03 (-0.21 to 0.14)	0.70	-0.03 (-0.21 to 0.15)	0.76	-0.001 (-0.18 to 0.18)	0.99	-0.01 (-0.20 to 0.18)	0.93
Effect of female sex at stroke on slope, per year	-0.03 (-0.31 to 0.25)	0.83	-0.05 (-0.33 to 0.24)	0.73	-0.09 (-0.37 to 0.20)	0.55	-0.05 (-0.35 to 0.25)	0.75
Effect of post-stroke systolic BP (per 10 mm Hg increase) on slope, per year	0.01 (-0.08 to 0.09)	0.91	NA	NA	NA	NA	-0.02 (-0.11 to 0.08)	0.70
Effect of post-stroke glucose (per 10 mg/dL increase) on slope, per year	NA	NA	0.001 (-0.04 to 0.04)	0.96	NA	NA	-0.01 (-0.06 to 0.04)	0.67
Effect of post-stroke LDL cholesterol (per 10 mg/dL increase) on slope, per year	NA	NA	NA	NA	0.05 (0.01 to 0.10)	0.03	0.05 (0.001 to 0.10)	0.05
**REGARDS Cohort:** Median (interquartile range) number of memory function assessments before stroke was 5 (3, 7) and after stroke was 3 (2, 3).								

© 2023 Levine DA et al. *JAMA Network Open.*

Abbreviations: BP=blood pressure. LDL=low-density lipoprotein. NA=not applicable.
a: All cognitive measures are set to a T-score metric (mean 50, SD 10); a 1-point difference represents a 0.1 SD difference in the distribution of cognition across the 4 cohorts. Higher cognitive scores indicate better performance.
b: Linear mixed-effects models included time since stroke, race, sex, age at time of stroke, education, income, medication for hypertension, diabetes, and high cholesterol, pre-stroke BMI, waist circumference, smoking status, physical activity, alcohol consumption per week, history of MI, history of atrial fibrillation, glomerular filtration rate, cohort study, pre-stroke mean memory, pre-stroke mean systolic BP, pre-stroke mean glucose, pre-stroke mean LDL, post-stroke mean systolic BP, post-stroke mean glucose, post-stroke mean LDL, age at time of stroke*time since stroke, sex*time since stroke, post-stroke mean systolic BP*time since stroke, post-stroke mean glucose*time since stroke, post-stroke mean LDL*time since stroke, anti-hypertensive medication use*time, anti-hyperglycemic medication use*time, and lipid lowering medication use*time. To consider correlation between longitudinal global cognition measures, we included random intercept and slope effect associated with participants. Glucose, LDL cholesterol, and systolic BP values are divided by 10 so that the parameter estimates refer to a 10-unit change in the variables. Each cognitive outcome is set to missing (censored) at the time of second expert-adjudicated incident stroke, death, loss to follow-up, or the end of follow-up, whichever occurs first. Models M1a, M1b, and M1c estimate the individual effect of post-stroke time-varying mean systolic BP, glucose, and LDL cholesterol on post-stroke memory decline with separate models. Model M1a includes a post-stroke time-varying mean systolic BP level by time interaction and post-stroke time-varying mean systolic BP. Model M1b includes a post-stroke time-varying mean glucose level by time interaction and post-stroke time-varying mean glucose. Model M1c includes post-stroke time-varying mean LDL cholesterol level by time interaction and post-stroke time-varying mean LDL cholesterol. Model M2 estimates the joint effect of post-stroke time-varying mean systolic BP, glucose, and LDL cholesterol on post-stroke memory decline. Model M2 includes the post-stroke time-varying mean systolic BP, glucose, and LDL cholesterol and their interactions with time.

© 2023 Levine DA et al. *JAMA Network Open.*

eTable 18. Sensitivity Analysis of Association of Post-Stroke Vascular Risk Factor Levels with Post-Stroke Global Cognition[ac] Decline with Estimated Fasting Glucose Levels: STROKE COG Study, 1971 to 2021

	**Model[b] M1a: Time-varying post-stroke systolic BP (n=982)**		**Model M1b: Time-varying post-stroke glucose (n=787)**		**Model M1c: Time-varying post-stroke LDL cholesterol (n=734)**		**Model M2: Joint time-varying post-stroke systolic BP, glucose, and LDL cholesterol (n=609)**	
**Coefficient**	**Estimate (95% CI)**	***P***	**Estimate (95% CI)**	***P***	**Estimate (95% CI)**	***P***	**Estimate (95% CI)**	***P***
Slope (change in cognition over time), per year	−0.46 (−0.65 to −0.27)	<0.001	-0.35 (-0.52 to -0.19)	<0.001	−0.41 (−0.59 to −0.22)	<0.001	-0.53 (-0.75 to -0.30)	<0.001
Effect of age at stroke (per 10-year increase) on slope, per year	−0.25 (−0.35 to −0.14)	<0.001	-0.19 (-0.31 to -0.08)	0.001	−0.21 (−0.32 to −0.10)	<0.001	-0.20 (-0.32 to -0.08)	0.001
Effect of female sex at stroke on slope, per year	−0.20 (−0.39 to −0.02)	0.03	-0.28 (-0.49 to -0.07)	0.008	−0.23 (−0.44 to −0.02)	0.03	-0.29 (-0.51 to -0.06)	0.01
Effect of post-stroke systolic BP (per 10 mm Hg increase) on slope, per year	0.03 (−0.01 to 0.08)	0.16	N/A	N/A	N/A	N/A	0.04 (-0.03 to 0.11)	0.22
Effect of post-stroke glucose (per 10 mg/dL increase) on slope, per year	N/A	N/A	-0.03 (-0.06 to 0.01)	0.19	N/A	N/A	-0.04 (-0.09 to -0.001)	0.043
Effect of post-stroke LDL cholesterol (per 10 mg/dL increase) on slope, per year	N/A	N/A	N/A	N/A	0.01 (−0.02 to 0.04)	0.50	0.008 (-0.03 to 0.04)	0.67

Abbreviations: BP=blood pressure. LDL=low-density lipoprotein. N/A=not applicable.

A: All cognitive measures are set to a T-score metric (mean 50, SD 10); a 1-point difference represents a 0.1 SD difference in the distribution of cognition across the 4 cohorts. Higher cognitive scores indicate better performance.

B: Linear mixed-effects models included time since stroke, race, sex, age at time of stroke, cohort, education, income, medication for hypertension, diabetes, and high cholesterol, pre-stroke body mass index, waist circumference, smoking status, physical activity, alcohol consumption per week, history of MI, history of atrial fibrillation, glomerular filtration rate, cohort study, pre-

© 2023 Levine DA et al. *JAMA Network Open.*

stroke mean executive function, pre-stroke mean systolic BP, pre-stroke mean glucose, pre-stroke mean LDL, post-stroke mean systolic BP, post-stroke mean glucose, post-stroke mean LDL, age at time of stroke*time since stroke, sex*time since stroke, post-stroke mean systolic BP*time since stroke, post-stroke mean glucose*time since stroke, post-stroke mean LDL*time since stroke, anti-hypertensive medication use*time, anti-hyperglycemic medication use*time, and lipid lowering medication use*time. To consider correlation between longitudinal global cognition measures, we included random intercept and slope effect associated with participants. Glucose, LDL cholesterol, and systolic BP values are divided by 10 so that the parameter estimates refer to a 10-unit change in the variables. Each cognitive outcome is set to missing (censored) at the time of second expert-adjudicated incident stroke, death, loss to follow-up, or the end of follow-up, whichever occurs first. Models M1a, M1b, and M1c estimate the Individual effect of post-stroke time-varying mean systolic BP, glucose, and LDL cholesterol on post-stroke executive function decline with separate models. Model M1a includes a post-stroke time-varying mean systolic BP level by time interaction and post-stroke time-varying mean systolic BP. Model M1b includes a post-stroke time-varying mean glucose level by time interaction and post-stroke time-varying mean glucose. Model M1c includes post-stroke time-varying mean LDL cholesterol level by time interaction and post-stroke time-varying mean LDL cholesterol. Model M2 estimates the joint effect of post-stroke time-varying mean systolic BP, glucose, and LDL cholesterol on post-stroke executive function decline. Model M2 includes the post-stroke time-varying mean systolic BP, glucose, and LDL cholesterol and their interactions with time.

C: Median (interquartile range) number of global cognition assessments before stroke was 4 (2-7) and after stroke was 3 (1-4).

© 2023 Levine DA et al. *JAMA Network Open.*

eTable 19. Sensitivity Analysis of Association of Post-Stroke Vascular Risk Factor Levels with Post-Stroke Executive Function[ac] Decline with Estimated Fasting Glucose Levels: STROKE COG Study, 1971 to 2021

	**Model[b] M1a: Time-varying post-stroke systolic BP (n=853)**		**Model M1b: Time-varying post-stroke glucose (n=667)**		**Model M1c: Time-varying post-stroke LDL cholesterol (n=609)**		**Model M2: Joint time-varying post-stroke systolic BP, glucose, and LDL cholesterol (n=495)**	
**Coefficient**	**Estimate (95% CI)**	***P***	**Estimate (95% CI)**	***P***	**Estimate (95% CI)**	***P***	**Estimate (95% CI)**	***P***
Slope (change in cognition over time), per year	−0.47 (−0.68 to −0.26)	<0.001	-0.31 (-0.50 to -0.12)	0.001	−0.35 (−0.55 to −0.15)	<0.001	-0.50 (-0.76 to -0.24)	<0.001
Effect of age at stroke (per 10-year increase) on slope, per year	−0.10 (−0.21 to 0.02)	0.09	-0.02 (-0.15 to 0.11)	0.77	−0.07 (−0.18 to 0.05)	0.24	-0.07 (-0.20 to 0.06)	0.27
Effect of female sex at stroke on slope, per year	−0.17 (−0.37 to 0.03)	0.10	-0.21 (-0.44 to 0.02)	0.08	−0.15 (−0.37 to 0.06)	0.16	-0.22 (-0.47 to 0.02)	0.07
Effect of post-stroke systolic BP (per 10 mm Hg increase) on slope, per year	−0.01 (−0.06 to 0.05)	0.84	N/A	N/A	N/A	N/A	0.003 (-0.07 to 0.08)	0.94
Effect of post-stroke glucose (per 10 mg/dL increase) on slope, per year	N/A	N/A	-0.003 (-0.05 to 0.04)	0.88	N/A	N/A	-0.01 (-0.05 to 0.04)	0.80
Effect of post-stroke LDL cholesterol (per 10 mg/dL increase) on slope, per year	N/A	N/A	N/A	N/A	−0.003 (−0.04 to 0.03)	0.87	-0.01 (-0.05 to 0.03)	0.57

Abbreviations: BP=blood pressure. LDL=low-density lipoprotein. N/A=not applicable.

A: All cognitive measures are set to a T-score metric (mean 50, SD 10); a 1-point difference represents a 0.1 SD difference in the distribution of cognition across the 4 cohorts. Higher cognitive scores indicate better performance.

B: Linear mixed-effects models included time since stroke, race, sex, age at time of stroke, cohort, education, income, medication for hypertension, diabetes, and high cholesterol, pre-stroke body mass index, waist circumference, smoking status, physical activity, alcohol consumption per week, history of MI, history of atrial fibrillation, glomerular filtration rate, cohort study, pre-stroke mean executive function, pre-stroke mean systolic BP, pre-stroke mean glucose, pre-stroke mean LDL, post-stroke mean systolic BP, post-stroke mean glucose, post-stroke mean LDL, age at time of stroke*time since stroke, sex*time since stroke, post-stroke mean systolic BP*time since stroke, post-stroke mean glucose*time since stroke, post-stroke mean LDL*time since stroke, anti-hypertensive medication use*time, anti-hyperglycemic medication use*time, and lipid lowering medication use*time. To consider correlation between longitudinal global cognition

© 2023 Levine DA et al. *JAMA Network Open.*

measures, we included random intercept and slope effect associated with participants. Glucose, LDL cholesterol, and systolic BP values are divided by 10 so that the parameter estimates refer to a 10-unit change in the variables. Each cognitive outcome is set to missing (censored) at the time of second expert-adjudicated incident stroke, death, loss to follow-up, or the end of follow-up, whichever occurs first. Models M1a, M1b, and M1c estimate the individual effect of post-stroke time-varying mean systolic BP, glucose, and LDL cholesterol on post-stroke executive function decline with separate models. Model M1a includes a post-stroke time-varying mean systolic BP level by time interaction and post-stroke time-varying mean systolic BP. Model M1b includes a post-stroke time-varying mean glucose level by time interaction and post-stroke time-varying mean glucose. Model M1c includes post-stroke time-varying mean LDL cholesterol level by time interaction and post-stroke time-varying mean LDL cholesterol. Model M2 estimates the joint effect of post-stroke time-varying mean systolic BP, glucose, and LDL cholesterol on post-stroke executive function decline. Model M2 includes the post-stroke time-varying mean systolic BP, glucose, and LDL cholesterol and their interactions with time.

C: Median (interquartile range) number of executive function assessments before stroke was 2 (1-4) and after stroke was 2 (1-3).

© 2023 Levine DA et al. *JAMA Network Open.*

eTable 20. Sensitivity Analysis of Association of Post-Stroke Vascular Risk Factor Levels with Post-Stroke Memory[ac] Decline with Estimated Fasting Glucose Levels: STROKE COG Study, 1971 to 2021

	**Model[b] M1a: Time-varying post-stroke systolic BP (n=929)**		**Model M1b: Time-varying post-stroke glucose (n=737)**		**Model M1c: Time-varying post-stroke LDL cholesterol (n=673)**		**Model M2: Joint time-varying post-stroke systolic BP, glucose, and LDL cholesterol (n=548)**	
**Coefficient**	**Estimate (95% CI)**	***P***	**Estimate (95% CI)**	***P***	**Estimate (95% CI)**	***P***	**Estimate (95% CI)**	***P***
Slope (change in cognition over time), per year	−0.22 (−0.42 to −0.01)	0.04	-0.25 (-0.43 to -0.06)	0.008	−0.24 (−0.45 to −0.03)	0.03	-0.30 (−0.56 to −0.03)	0.03
Effect of age at stroke (per 10-year increase) on slope, per year	0.02 (−0.10 to 0.14)	0.73	0.02 (-0.11 to 0.15)	0.74	−0.02 (−0.16 to 0.11)	0.72	0.01 (−0.14 to 0.16)	0.88
Effect of female sex at stroke on slope, per year	−0.29 (−0.50 to −0.07)	0.01	−0.29 (−0.52 to −0.06)	0.02	−0.33 (−0.57 to −0.08)	0.009	−0.27 (−0.54 to −0.01)	0.045
Effect of post-stroke systolic BP (per 10 mm Hg increase) on slope, per year	0.03 (−0.02 to 0.08)	0.24	N/A	N/A	N/A	N/A	0.03 (-0.05 to 0.11)	0.45
Effect of post-stroke glucose (per 10 mg/dL increase) on slope, per year	N/A	N/A	0.000 (−0.04 to 0.04)	0.99	N/A	N/A	-0.01 (−0.06 to 0.04)	0.67
Effect of post-stroke LDL cholesterol (per 10 mg/dL increase) on slope, per year	N/A	N/A	N/A	N/A	−0.003 (−0.04 to 0.03)	0.87	0.01 (−0.03 to 0.05)	0.80

Abbreviations: BP=blood pressure. LDL=low-density lipoprotein. N/A=not applicable.

A: All cognitive measures are set to a T-score metric (mean 50, SD 10); a 1-point difference represents a 0.1 SD difference in the distribution of cognition across the 4 cohorts. Higher cognitive scores indicate better performance.

© 2023 Levine DA et al. *JAMA Network Open.*

B: Linear mixed-effects models included time since stroke, race, sex, age at time of stroke, cohort, education, income, medication for hypertension, diabetes, and high cholesterol, pre-stroke body mass index, waist circumference, smoking status, physical activity, alcohol consumption per week, history of MI, history of atrial fibrillation, glomerular filtration rate, cohort study, pre-stroke mean executive function, pre-stroke mean systolic BP, pre-stroke mean glucose, pre-stroke mean LDL, post-stroke mean systolic BP, post-stroke mean glucose, post-stroke mean LDL, age at time of stroke*time since stroke, sex*time since stroke, post-stroke mean systolic BP*time since stroke, post-stroke mean glucose*time since stroke, post-stroke mean LDL*time since stroke, anti-hypertensive medication use*time, anti-hyperglycemic medication use*time, and lipid lowering medication use*time. To consider correlation between longitudinal global cognition measures, we included random intercept and slope effect associated with participants. Glucose, LDL cholesterol, and systolic BP values are divided by 10 so that the parameter estimates refer to a 10-unit change in the variables. Each cognitive outcome is set to missing (censored) at the time of second expert-adjudicated incident stroke, death, loss to follow-up, or the end of follow-up, whichever occurs first. Models M1a, M1b, and M1c estimate the individual effect of post-stroke time-varying mean systolic BP, glucose, and LDL cholesterol on post-stroke executive function decline with separate models. Model M1a includes a post-stroke time-varying mean systolic BP level by time interaction and post-stroke time-varying mean systolic BP. Model M1b includes a post-stroke time-varying mean glucose level by time interaction and post-stroke time-varying mean glucose. Model M1c includes post-stroke time-varying mean LDL cholesterol level by time interaction and post-stroke time-varying mean LDL cholesterol. Model M2 estimates the joint effect of post-stroke time-varying mean systolic BP, glucose, and LDL cholesterol on post-stroke executive function decline. Model M2 includes the post-stroke time-varying mean systolic BP, glucose, and LDL cholesterol and their interactions with time.

C: Median (interquartile range) number of memory assessments before stroke was 3 (2-5) and after stroke was 2 (1-3).

© 2023 Levine DA et al. *JAMA Network Open.*

eReferences

1. Griffith L, van den Heuvel E, Fortier I, et al. AHRQ Methods for Effective Health Care. *Harmonization of Cognitive Measures in Individual Participant Data and Aggregate Data Meta-Analysis*. Rockville (MD): Agency for Healthcare Research and Quality (US); 2013. Report No. 13-EHC040-EF.
2. Briceño EM, Gross AL, Giordani BJ, et al. Pre-Statistical Considerations for Harmonization of Cognitive Instruments: Harmonization of ARIC, CARDIA, CHS, FHS, MESA, and NOMAS. *J Alzheimers Dis*. 2021;83(4):1803-1813. PMCID:PMC8733857.
3. Asparouhov T, Muthén B. Plausible Values for Latent Variables Using Mplus: Technical Report. Updated 21 August. Accessed 29 April, 2019. http://www.statmodel.com/download/Plausible.pdf
4. Muthén LK, Muthén BO. *Mplus User's Guide, 8th Edition*. 8th ed. Muthén & Muthén; 2017. Accessed 5 January 2022. https://www.statmodel.com/download/usersguide/MplusUserGuideVer_8.pdf
5. Kolen M, Brennan R. *Test Equating: Methods and Practices*. New York: Springer; 1995.
6. Gross AL, Jones RN, Fong TG, Tommet D, Inouye SK. Calibration and validation of an innovative approach for estimating general cognitive performance. *Neuroepidemiology*. 2014;42(3):144-153. PMCID:PMC3988278.
7. Levine DA, Gałecki AT, Langa KM, et al. Blood pressure and cognitive decline over 8 years in middle-aged and older Black and White Americans. *Hypertension*. 2019;73(2):310-318. PMCID:PMC6450556.
8. Lau KK, Li L, Simoni M, Mehta Z, Kuker W, Rothwell PM. Long-term premorbid blood pressure and cerebral small vessel disease burden on imaging in transient ischemic attack and ischemic stroke. *Stroke*. 2018;49(9):2053-2060.
9. Franklin SS, Jacobs MJ, Wong ND, L'Italien GJ, Lapuerta P. Predominance of isolated systolic hypertension among middle-aged and elderly US hypertensives: analysis based on National Health and Nutrition Examination Survey (NHANES) III. *Hypertension*. 2001;37(3):869-874.
10. Pool LR, Ning H, Wilkins J, Lloyd-Jones DM, Allen NB. Use of Long-term Cumulative Blood Pressure in Cardiovascular Risk Prediction Models. *JAMA Cardiol*. 2018;3(11):1096-1100. PMCID:PMC6583053.
11. Levine DA, Gross AL, Briceno EM, et al. Association between blood pressure and later-life cognition among Black and White individuals. *JAMA Neurology*. 2020;77(7):810-819. PMCID:PMC7154952.
12. Crane PK, Walker R, Hubbard RA, et al. Glucose Levels and Risk of Dementia. *New England Journal of Medicine*. 2013;369(6):540-548.
13. Chun HY, Ford A, Kutlubaev MA, Almeida OP, Mead GE. Depression, Anxiety, and Suicide After Stroke: A Narrative Review of the Best Available Evidence. *Stroke*. 2022;53(4):1402-1410.
14. Kauhanen M, Korpelainen JT, Hiltunen P, et al. Poststroke depression correlates with cognitive impairment and neurological deficits. *Stroke*. 1999;30(9):1875-1880.
15. Callahan CM, Unverzagt FW, Hui SL, Perkins AJ, Hendrie HC. Six-item screener to identify cognitive impairment among potential subjects for clinical research. *Med Care*. 2002;40(9):771-81.

© 2023 Levine DA et al. *JAMA Network Open.*
