# Supplementary material for: Associations Between Vascular Risk Factor Levels and Cognitive Decline Among Stroke Survivors
Source: JAMA Netw Open. 2023 May 17;6(5):e2313879. doi: 10.1001/jamanetworkopen.2023.13879 (PMC10193182; doi:10.1001/jamanetworkopen.2023.13879)
Supplement: Supplement 2. — Data Sharing Statement [file jamanetwopen-e2313879-s002.pdf]

# Data Sharing Statement

Levine. Associations Between Vascular Risk Factor Levels and Cognitive Decline Among Stroke Survivors. *JAMA Netw Open*. Published May 17, 2023.
doi:10.1001/jamanetworkopen.2023.13879

**Data**
**Data available:** Yes
**Data types:** Other (please specify)
**Additional Information:** Deidentified participant data if with the approval of a proposal by the individual cohorts and principal investigator and with signed data use agreements between all relevant institutions.
**How to access data:** deblevin@umich.edu
**When available:** With publication

**Supporting Documents**
**Document types:** Statistical/analytic code
**How to access documents:** deblevin@umich.edu
**When available:** With publication

**Additional Information**
**Who can access the data:** Researchers whose proposed use of the data have been approved by the individual cohorts and principal investigator and who have signed data use agreements between all relevant institutions.
**Types of analyses:** For specified purposes approved by the individual cohorts and principal investigator
**Mechanisms of data availability:** Data will be made available with the approval of a proposal by the individual cohorts and principal investigator and with signed data use agreements between all relevant institutions.
